# Inter-comparison of marine microbiome sampling protocols

**DOI:** 10.1038/s43705-023-00278-w

**Published:** 2023-08-19

**Authors:** Francisco Pascoal, Maria Paola Tomasino, Roberta Piredda, Grazia Marina Quero, Luís Torgo, Julie Poulain, Pierre E. Galand, Jed A. Fuhrman, Alex Mitchell, Tinkara Tinta, Timotej Turk Dermastia, Antonio Fernandez-Guerra, Alessandro Vezzi, Ramiro Logares, Francesca Malfatti, Hisashi Endo, Anna Maria Dąbrowska, Fabio De Pascale, Pablo Sánchez, Nicolas Henry, Bruno Fosso, Bryan Wilson, Stephan Toshchakov, Gregory Kevin Ferrant, Ivo Grigorov, Fabio Rocha Jimenez Vieira, Rodrigo Costa, Stéphane Pesant, Catarina Magalhães

**Affiliations:** 1 Interdisciplinary Centre of Marine and Environmental Research, University of Porto, Terminal de Cruzeiros do Porto de Leixões, Av. General Norton de Matos s/n, 4450-208 Porto, Portugal; 2 Departamento de Biologia, Faculdade de Ciências, Universidade do Porto, rua do Campo Alegre s/n, 4169– 007 Porto, Portugal; 3 Integrative Marine Ecology Department, Stazione Zoologica Anton Dohrn, Naples, Italy; 4 Institute for Biological Resources and Marine Biotechnologies, National Research Council (IRBIM-CNR), Largo Fiera della Pesca 2, 60125 Ancona, Italy; 5 Faculty of Computer Science, Dalhousie University, Halifax, NS Canada; 6 Génomique Métabolique, Genoscope, Institut François Jacob, CEA, CNRS, Univ Evry, Université Paris-Saclay, 2 Rue Gaston Crémieux, 91057 Evry, France; 7 Sorbonne Université, CNRS, Laboratoire d’Écogéochimie des Environnements Benthiques (LECOB), Observatoire Océanologique de Banyuls, Banyuls-sur-Mer, France; 8 Marine & Environmental Biology, Department of Biological Sciences, University of Southern California (USC), Los Angeles, CA USA; 9 EMBL’s European Bioinformatics Institute (EMBL-EBI), Wellcome Genome Campus, Hinxton, Cambridgeshire CB10 1SD UK; 10 National Institute of Biology, Marine Biology Station Piran, Piran, Slovenia; 11 Lundbeck Foundation GeoGenetics Centre, GLOBE Institute, University of Copenhagen, Copenhagen, Denmark; 12 Department of Biology, University of Padua, Via U. Bassi 58/B, 35131 Padua, Italy; 13 Institute of Marine Sciences (ICM), CSIC. Passeig Marítim de la Barceloneta, 37-49, ES08003 Barcelona, Spain; 14 Department of Life Sciences, University of Trieste, Trieste, Italy; 15 Bioinformatics Center, Institute for Chemical Research, Kyoto University, Gokasho, Uji Japan; 16 Department of Marine Ecology, Institute of Oceanology Polish Academy of Sciences, Sopot, Poland; 17 Sorbonne Université, CNRS, Station Biologique de Roscoff, AD2M ECOMAP, UMR 7144, Roscoff, France; 18 CNRS, FR2424, ABiMS, Station Biologique de Roscoff, Sorbonne Université, Roscoff, France; 19 Department of Biosciences, Biotechnologies and Environment, University of Bari, 70126 Bari, Italy; 20 Department of Biology, John Krebs Field Station, University of Oxford, Wytham, OX2 8QJ UK; 21 Kurchatov Center for Genome Research, Moscow, Russia; 22 Rannsóknir og nýsköpun/Research & Innovation, Matís, Reykjavík, Iceland; 23 Technical University of Denmark, National Institute of Aquatic Resources, Kgs. Lyngby, Denmark; 24 Départment de Biologie, École Normale Supérieure, Paris, France; 25 Department of Bioengineering, Instituto Superior Técnico, University of Lisbon, Av. Rovisco Pais, 1049-001 Lisbon, Portugal; 26 Institute for Bioengineering and Biosciences (iBB) and i4HB—Institute for Health and Bioeconomy, Instituto Superior Técnico, University of Lisbon, Av. Rovisco Pais, 1049-001 Lisbon, Portugal

**Keywords:** Metagenomics, Microbial ecology

## Abstract

Research on marine microbial communities is growing, but studies are hard to compare because of variation in seawater sampling protocols. To help researchers in the inter-comparison of studies that use different seawater sampling methodologies, as well as to help them design future sampling campaigns, we developed the EuroMarine Open Science Exploration initiative (EMOSE). Within the EMOSE framework, we sampled thousands of liters of seawater from a single station in the NW Mediterranean Sea (Service d'Observation du Laboratoire Arago [SOLA], Banyuls-sur-Mer), during one single day. The resulting dataset includes multiple seawater processing approaches, encompassing different material-type kinds of filters (cartridge membrane and flat membrane), three different size fractionations (>0.22 µm, 0.22–3 µm, 3–20 µm and >20 µm), and a number of different seawater volumes ranging from 1 L up to 1000 L. We show that the volume of seawater that is filtered does not have a significant effect on prokaryotic and protist diversity, independently of the sequencing strategy. However, there was a clear difference in alpha and beta diversity between size fractions and between these and “whole water” (with no pre-fractionation). Overall, we recommend care when merging data from datasets that use filters of different pore size, but we consider that the type of filter and volume should not act as confounding variables for the tested sequencing strategies. To the best of our knowledge, this is the first time a publicly available dataset effectively allows for the clarification of the impact of marine microbiome methodological options across a wide range of protocols, including large-scale variations in sampled volume.

## Introduction

The characterization of microbial life on Earth has become a topic of transdisciplinary interest. Indeed, it is now recognized that acquiring knowledge in microbial ecology across multiple biomes is crucial to develop a deeper understanding of life from cells to ecosystems. As a result, massive international collaborative research projects have focused on microbiomes associated with humans [1, 2], corals [3], seagrass (https://seagrassmicrobiome.org/protocols/) or sponges [4, 5]. In addition, over the past 20 years, global coordinated marine planktonic microbiome sampling initiatives have been launched, such as the Global Ocean Sampling (2003–2010) [6, 7], the International Census of Marine Microbes (ICoMM) [8], Malaspina 2010 Circumnavigation Expedition [9] and Tara Ocean expeditions (2009–2012) [10], together with census programs such as the Earth Microbiome program [11, 12], and the Micro B3-led Ocean Sampling Day (OSD) [13]. Details on advances and perspectives on global ocean microbial ecology, their relevance and future challenges have been extensively reviewed elsewhere [14].

The current massive effort to study the world’s microbiomes has given rise to multiple standardization initiatives including the use of common protocols for sampling the microbiome of different environments and host tissues, and of common sequencing procedures. Relevant initiatives with methodological standardization efforts include, for example, OSD [13], Earth Microbiome [15], European Marine Omics Biodiversity Observatory Network (EMO BON) [16] and Metagenomics for Human Intestinal Tract (MetaHIT) [17].

The large-scale analysis of free-living and host-associated microbiomes constitutes a huge step forward in understanding microbes-animal [3, 5, 18, 19] and microbes-plant [20] interactions, as well as the structure, function and diversity of microbial communities in diverse Earth habitats [21]. However, gaps need to be filled to better standardize and harmonize the best practice and strategies to sample, describe and study microbial diversity. In particular, in ocean microbiome studies, it is known that the estimation of microbial richness depends on several factors, including the marker genes and primers used for metabarcoding [22], different DNA extraction protocols [23, 24], the sequencing depth and genomic approach (amplicon sequencing vs metagenome sequencing) and clustering criteria [25]. Although the sampling strategy is recognized to influence estimations of microbial plankton diversity [25], there is a lack of studies designed to systematically test the effect of methodological variables on the sampling procedures to study ocean microbiome diversity and taxonomic composition. These studies are crucial to design precise protocols to sample the entire size-range of marine microbial communities [26].

To study marine microbes, it is necessary to collect seawater and then to concentrate the cells through filtration. The filtered volume is usually in the range of 0.5 L, 1 L, 3–10 L and 100 L (e.g., [7, 27–29]) or until the filter gets clogged, depending on sediment particles present, organic matter detritus, cell biomass and/or growing microalgae [30]. Thus, according to the trophic status of the system, its hydrographic conditions and the proximity to terrestrial runoff sources, different volumes of water might be needed, or a pre-filtration step added. It is uncommon to use volume in the range of the microliters, but it has been used, for example, to test bacterium-bacterium interactions at millimeter scales [31]. It is often possible to find variations in the filtered volume within the same study, for example, because of on-site methodological constraints, e.g., [29], or for samples intended for different purposes, like DNA and RNA collection, e.g., [32]. In addition, there are two main types of filters widely used by the scientific community: (1) cartridge membrane filters, with a pore size of 0.22 μm; and (2) flat membrane filters, which can also be used for size fractionation. The flat membrane filters also differ in the material they comprise (polyethersulfone, polycarbonate, cellulose, etc.), which affects their properties [33]. A previous study compared amplicon sequencing results of 16 S, 18 S and 12 S rRNA genes for five flat membrane filters with different compositions, and found no significant differences [34]. Another study, however, highlighted that different filter materials and DNA extraction protocols can introduce false negative detection of microeukaryotic operational taxonomic units (OTUs) and underestimate diversity [35]. Size fractionation is used to separate microbial cells by size [36, 37], thus selecting prokaryotes from larger microeukaryotes and discriminating free-living cells from particle attached ones. Classically, samples are divided into picoplankton (0.2–3 µm), nanoplankton (3–20 µm) and microplankton (20–200 µm) size fractions, based on the historic division of planktonic size fractions [38]. However, there are variations across different studies, for example selecting for picoplankton in the 0.8–3 µm range [39]. Previous studies have addressed the distribution of microeukaryotes across size fractions, observing that size fractionation can introduce artifacts from cell collapse and subsequent retention on smaller sized fractions [40]. Additionally, variables such as the shape and life cycle stage of protists can result in the identification of the same species across different size fractions, as observed for diatoms [39].

Within the frame of the EMOSE 2017 initiative, we sampled water from a coastal site of the NW Mediterranean Sea (SOLA), and constructed a unique dataset of deeply-sequenced metagenomes and 16 S/18 S rRNA gene amplicons (MetaB16SV4V5 and MetaB18SV9). To our knowledge, this dataset represents the largest sequencing effort ever conducted at a single site on a single day. The experiment was designed to compare different filtration volumes, filter types, size fractionations and sequencing strategies earlier used in some of the most relevant global ocean initiatives, i.e., Tara Oceans [10], Malaspina [9], and the OSD [13].

The EMOSE sampling was designed to evaluate how microbial diversity estimates change with (i) changing volume of filtered seawater, (ii) different filter types (10 L of water on 0.22 µm cartridge versus flat membrane filters), (iii) whole water filtration versus size fractionation (10 L of water on flat membrane filters); (iv) different size fractions (100 L through 20 µm, 3 µm and 0.22 µm pore size filters); and (v) a single 2.5 L filter versus 4 pooled filters of 2.5 L (0.22 µm pore size, whole water, cartridge membrane). All of the aforementioned comparisons were considered independently for MetaB16SV4V5, MetaB18SV9 and metagenomics, for prokaryotes and protists. Note that several studies include viruses and fungi in their definition of “microbiome”, e.g., [14]. For the purposes of this article, we are only considering prokaryotes and unicellular eukaryotes, unless stated otherwise.

The size, uniqueness and accessibility of the EMOSE dataset have great potential to help clarify the impact of methodological differences between studies and to contribute to the standardization of applied procedures. It is also open source and freely available and will allow for further investigations beyond the scope of this study.

## Materials and methods

### Seawater sampling

Sampling took place at a single location, the SOLA station (42°29'300 N – 03°08’700 E) in the bay of Banyuls-sur-Mer (NW Mediterranean Sea), aboard the research vessel RV Nereis II from the Oceanological Observatory of Banyuls-sur-Mer (OOB). A total of 75 carboys of 20 L were collected on a single day (2017–05–30) from subsurface water (3 m depth) using a high-volume well pump for about 45 min. Carboy containers were divided into different seawater sampling strategies to minimize the sampling bias of ship drifting and diurnal variation. The carboy containers used to store the seawater were washed with diluted bleach (10% v/v) the day before and thoroughly rinsed twice with sample water before being filled.

**Fig. 1:**
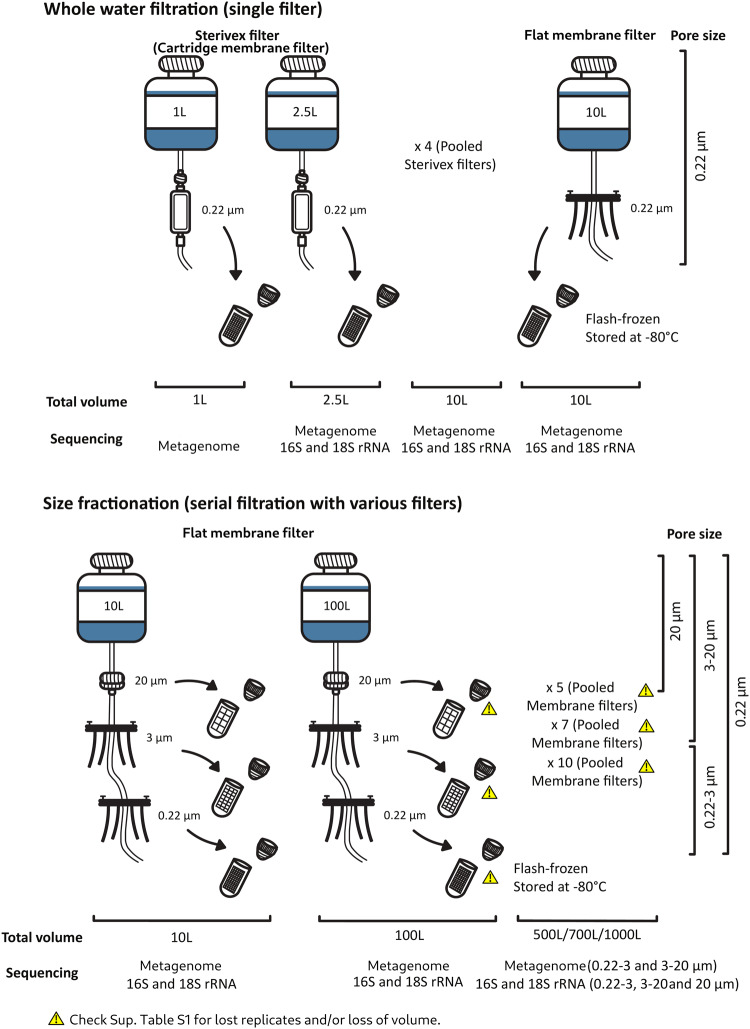
Schematic representation of sampling campaign. An attempt was made to have at least three replicates of each step, however, some steps lost replicates and/or volume, those situations are highlighted with an attention sign in this figure. For more details on replicates, see Supplementary Table S1.

Water filtration was performed following several seawater sampling protocols (Fig. 1 and Supplementary Table S1). For the analysis of the total volume of water collected: 1 L was filtered through a single cartridge membrane filter of 0.22 µm; 2.5 L through a single cartridge membrane filter of 0.22 µm; 10 L through a single flat membrane filter of 0.22 µm. For the size fractions analysis, microbes were collected by serial filtration through three filters of decreasing pore sizes, according to the following procedures: 10 L through a mesh filter of 20 µm followed by 3 µm and 0.22 µm flat membrane filters; 100 L through a mesh filter of 20 µm, followed by flat membrane filters of 3 µm and 0.22 µm. All filters were flash-frozen in liquid nitrogen and stored in a freezer at −80 °C.

The protocols for cartridge membrane filtration were performed with the use of the Sterivex cartridge membrane filter unit (Product Code SVGPB1010, Millipore) with a polyethersulfone membrane, while the protocols for membrane filtration of whole water community (>0.22 µm) and of the 0.22 µm to 3 µm size fractions used 142 mm diameter polyethersulfone Express Plus membrane filters (Product Code GPWP14250, Millipore). For the 3 µm to 20 µm fractionations, 142 mm diameter polycarbonate membrane filters were used (Product Code TSTP14250, Millipore). As for the large size fractions (>20 µm), the 47 mm diameter nylon mesh filter was used instead (referred to as flat membrane from here on).

Following the sampling scheme in Fig. 1 and Supplementary Table S1, the present study analyzed 79 seawater samples (*n* = 157, including successful replicates) according to commonly applied methodologies, expanded to include (and compare) the use of various filter types, different volumes of filtered water, and the division of plankton based on its size (Supplementary Table S1). Note that some replicates were lost during seawater filtration, DNA extraction and/or sequencing, we refer the reader to Supplementary Table S1 for information on the number of replicates successfully obtained. Furthermore, some samples were discarded during rarefaction due to low number of reads (more details below). The metadata relative to each sample, including the sub-samples used for pooling of larger volume samples, are described in detail in Supplementary Table S2.

### DNA extraction, amplification and sequencing

A full description of the following protocols for molecular data production is available in Alberti et al. [41].

Briefly, DNA extraction began by cryo-grinding (SPEX SamplePrep 6870 Freezer/Mil, Fisher Scientific) the filters with lysis buffer and BSH, followed by a filter column XL (Macherey-Nagel), with lysis buffer and BSH. Purification was done with Nucleospin RNA II, with 1 volume of filtrate and equal volume of ethanol (70% v/v), followed by elution of DNA with nucleopsin buffer set (Macherey Nagel). DNA was quantified by a dsDNA-specific fluorimetric quantitation method using Qubit 2.0 Fluorometer (Thermo Fisher Scientific) with Qubit dsDNA BR (Broad-Range) and HS (High-Sensitivity) Assays and stored at −20 °C.

For DNA shotgun sequencing (metagenome), the library preparation was performed using a protocol for low DNA input. 10 ng of total DNA were sonicated and sequencing libraries prepared using NEBNext Ultra II DNA Library Prep Kit for Illumina (New England BioLabs). Fragments were end-repaired, 3’-adenylated and NEXTflex DNA barcoded adaptors were added as per the manufacturer’s instructions. After two consecutive 1x Ampure XP (Fisher Scientific) clean ups, the ligated products were PCR-amplified with the NEBNext Ultra II Q5 Master Mix (included in the kit), followed by 0.8x AMPure XP purification. Prepared libraries were first quantified by Qubit dsDNA HS Assay measurement. A size-profile analysis was then conducted in an Agilent 2100 Bioanalyzer (Agilent Technologies, Santa Clara, CA, USA) and by qPCR with the KAPA Library Quantification Kit for Illumina Libraries (Kapa Biosystems, Wilmington, MA, USA) on an MXPro instrument (Agilent Technologies, Santa Clara, CA, USA). Libraries were subjected to Illumina sequencing on a HiSeq 4000 instrument (Illumina), with 150 bp paired-end reads layout. Three samples were sequenced with the Rapid HiSeq 4000 instrument, again using the 150 bp paired-end reads layout.

For amplicon sequencing of the V9 hypervariable region of the 18 S rRNA gene (MetaB18SV9), DNA was amplified with the primers 1389 F 5’- TTGTACACACCGCCC -3’ and 1510 R 5’- CCTTCYGCAGGTTCACCTAC -3’ [42]. Three PCR reactions per sample were set up using PCR mixtures (25 μl final volume) containing 5 to 10 ng of total DNA template with 0.35 μM final concentration of each primer, 3% of DMSO and 1X Phusion Master Mix. PCR amplifications were performed as follow: 98 °C for 30 s; 25 cycles of 10 s at 98 °C, 30 s at 57 °C, 30 s at 72 °C; and 72 °C for 10 min. PCR products were then pooled and purified by 1.8x AMPure XP beads (Beckman Coulter Genomics) cleanup. The PCR products varied in length from 170–180 bp. A negative control (Nuclease-free water) was included. All libraries were prepared using the NEBNext DNA Modules Products and NextFlex DNA barcodes with 100 ng of purified PCR product as input and sequenced using HiSeq 2500 Rapid (Illumina) machine (150 bp paired-end reads). Three samples were sequenced with the MiSeq instrument.

For amplicon sequencing of the V4-V5 hypervariable regions of the 16 S rRNA gene (MetaB16SV4V5), DNA was amplified with the primers 515 F (Forward: 5’-GTGYCAGCMGCCGCGGTAA-3’) and 926 R (Reverse: 5’-CCGYCAATTYMTTTRAGTTT-3’) [22, 43–45]. For each sample, six reactions were used using the same PCR mixtures as above, with thermal cycling of 30 s at 98 °C, followed by 37 cycles of 10 s at 98 °C, 30 s at 53 °C and 30 s at 72 °C, ending with a final 10 min at 72 °C. Please be aware that these PCR conditions were changed from those in ref. [23], because the polymerase used was different. PCR products were then pooled and purified by 1x AMPure beads. The PCR product size varied from 300 bp to 700 bp. All libraries were prepared using the NEBNext DNA Modules Products and NextFlex DNA barcodes with 250 ng of purified PCR product as input. In parallel, one negative control (water) and 16 mock communities were used: 8 mock communities of prokaryotes and 8 of eukaryotes (provided by the Jed Fuhrman laboratory, described in Parada et al. [22] and Yeh et al. [46]. After AMPure XP purification (1 volume) and quantification by Qubit fluorometric measurement (HS assay), equimolar pools of amplified libraries were run on a 2% (w/v) agarose gel to select 500–650 bp gel slices (amplicon size increased by Illumina adapters). This sizing step separated the prokaryotic 16 S amplicons from the eukaryotic amplification products, which were not sequenced in this study. The sized library was finally purified using the Nucleospin Extract II DNA purification kit. Sequencing was carried out on a HiSeq 2500 Rapid machine, with 250 bp paired-end reads. Eight samples were sequenced, together with respective mock communities, using a MiSeq machine instead, with a 2 × 300 bp paired-end mode. Please note that sequencing results for samples relative to read length size division and mock communities were made publicly available, but were not reported in this article.

### Bioinformatics processing of raw sequences

The FASTQ files of the produced sequences were submitted to the European Nucleotide Archive (ENA: Accession PRJEB87662), where they are publically available. Raw reads were processed by the MGnify platform [47]. More specifically, Version 5.0 was used for the amplicon data (MataB16SV4V5 and MetaB18SV9), while Version 4.1 was used for the metagenomic data. Briefly, forward and reverse reads were merged with SeqPrep v1.2, quality filtered with Trimmomatic v0.36, reads with less than 100 bp and with more than 10% bp ambiguity were removed. Infernal v1.1.2 [48] was used together with Rfam 13.0 [49] for identification of SSU rRNA genes. Amplicon reads were directly attributed taxonomic lineages using pre-computed operational taxonomic units with MAPSeq v1.2.3 [50] and SILVA database v132 [51]. For shotgun metagenomics data, mOTU2 [52] were used with SILVA database v132 [51] for taxonomic lineages. The number of reads at each step, for each sample and the respective accession numbers and links are available in Supplementary Table S3. Despite the recent revision in the taxonomy of microorganisms at the phylum level, and hence substantial nomenclature modifications [53], we used the taxonomic information as provided by the MGnify platform after the SILVA database v132 [51].

### Abundance tables pre-processing

The tables with abundance per taxonomic lineage and sample were directly transferred from the MGnify platform to the R software environment v3.6.3 [54]. We started by dividing the sequencing runs into prokaryotes and protists. Specifically, the MetaB16SV4V5 was filtered to include taxonomic lineages attributed to prokaryotes. We removed any taxonomic lineages attributed to organelles (mitochondria and chloroplasts). Using the same reasoning, for the MetaB18SV9 approach, we focused on protists instead and excluded any taxonomic lineages assigned to prokaryotic, metazoan, fungi, or viridiplantae taxonomy. Metagenomic data were subdivided into a prokaryotic dataset and a protist dataset, because sequencing all DNA without primer bias allows to identify either biological groups. Notwithstanding, we considered them to be independent biological groups and reasoned that it would be more informative to separate them. This separation was performed after removal of any taxonomic lineages associated with organelles, metazoan, fungi and viridiplantae. For either approach, the taxonomic lineages with NA taxonomy at Phylum level were discarded. The abundance tables were then downloaded for manual curation of taxonomy to add a “fake rank” column with relevant taxonomy of both prokaryotes and protists. For protists, we focused on Phyla and Classes of most interest, while for prokaryotes the “fake rank” included all phyla, but Proteobacteria was subdivided by class level.

After taxonomy curation, we removed singleton taxonomic lineages from amplicon sequencing approaches (MetaB16SV4V5 and MetaB18SV9). The sequencing depth was not homogeneous between the variables that we intend to directly compare, which could result in biased comparisons of diversity, thus we decided to apply rarefaction after removing singletons. The threshold for rarefaction was considered individually for each sequencing approach (MetaB16SV4V5, MetaB18SV9, and metagenomes) and biological group (prokaryotes and protists), because they are independent and represent different orders of magnitude of sequencing depth. Additionally, the specific rarefaction threshold applied should counterbalance the cost of losing too many high quality reads against losing too many valid samples. Specifically, samples from MetaB16SV4V5 were rarefied to 250,000 reads and three samples were discarded; samples from MetaB18SV9 were rarefied to 100,000 reads and six samples were discarded; samples from metagenomes, considering only the prokaryotic taxonomic lineages, were rarefied to 10,000 reads and 15 samples were discarded; and samples from metagenomes, considering protist taxonomic lineages, were rarefied to 1000 reads and four samples were discarded. The samples discarded are available in Supplementary Table S4.

### Statistical analysis

All statistical analyses were performed in the R software environment v3.6.3 [54]. Alpha and beta diversity metrics were calculated using the vegan v2.5.7 package [55], all figures, except Fig. 1, were produced with the ggplot2 v3.4.0 package [56] or base R. Statistical tests and their assumptions were tested using the rstatix v0.7.0 package [57] and followed the guidelines for best practices proposed in [57]. The alpha diversity metric used was the total number of taxonomic lineages in a given sample, i.e., species richness. We decided to use a single alpha diversity metric to simplify readability of the results and selected species richness because it is the most straightforward alpha diversity metric. This alpha diversity metric allows us to assess the direct output of the methodologies compared, making it the most general purpose one. We acknowledge that using a single alpha diversity metric would be very reductive in an environmental research setting. However, we did not make inferences on the ecology of the system and several alpha diversity metrics could be redundant with each other. For a sanity check, we verified that the Shannon index would probably get similar results, while the Simpson index could provide different results, based on correlation analysis (Supplementary Fig. S1).

We decided to use nonparametric statistical tests to compare species richness between variables, because of the limited number of samples for specific comparisons. For the comparison of two independent groups, we used the Mann–Whitney test [58, 59]. For more than two independent groups, we used the Kruskal–Wallis test [59]. Statistically significant results from Kruskal–Wallis were followed by the Dunn *post-hoc* test [60]. The tests were performed for comparisons with at least three replicate samples per independent group. For each test, we specified if the *p* value was significant or not (alpha = 0.05), after adjusting with the Bonferroni method for multiple comparisons [61].

To describe beta diversity, we compared the dissimilarity between methodological variables. For that purpose, we used Bray–curtis dissimilarity matrices and visualized the distance between samples with ordination plots, specifically nMDS, with the metaMDS function of the vegan package [55]. Significance values were calculated by PERMANOVA with adonis2 function and homogeneity of variance was verified with betadisper function, both from the vegan package [55]. Finally, significance of distance to centroid was accessed with Tukey test, with base R functions.

## Results

### Environmental communities

For the MetaB16SV4V5 sequencing results (*n* = 60), we initially obtained between 714,103 and 2,841,890 raw reads per sample (median = 1,462,584 reads, IQR = 267,472 reads). The final number of high quality reads attributed to taxonomic lineages ranged between 169,945 and 1,517,860 reads per sample (median = 660 878 reads, IQR = 438 932 reads). Thus, between 13.38% and 72.47% of reads were kept after the quality filtering and processing into taxonomic lineages (median = 50.69%, IQR = 30.47%). MetaB18SV9 sequencing results (*n* = 47) obtained between 670,823 and 3,876,463 raw reads per sample (median = 1,322,612 reads, IQR = 319,425 reads). From those reads, the final, high quality reads processed into taxonomic lineages ranged between 66,912 and 1,889,838 reads per sample (median = 1,733,379 reads, IQR = 348,105 reads). Thus, between 6.21% and 60.33% of reads were kept after the quality filtering and processing into taxonomic lineages (median = 13.05%, IQR = 26.13%). For metagenomes (*n* = 50), we obtained between 36 573,050 and 123,310,150 raw reads per sample (median = 58,122,461 reads, IQR = 12,863,822 reads). A minor fraction of the metagenome reads was used for taxonomic identification. Specifically, for prokaryotes, the 16S rRNA reads ranged from 628 to 98,749 (median = 23,376 reads, IQR = 43,212 reads), thus corresponding to a ratio between 0.0011% and 0.0976% (median = 0.0394%, IQR = 0.0664%) of final vs initial raw reads. For protists, the range of final metagenome 18S rRNA reads used was between 396 and 9160 reads (median = 2568 reads, IQR = 2807 reads), thus corresponding to a ratio between 0.0006% and 0.0121% (median = 0.0051%, IQR = 0.0046%). The values of the sequencing results are summarized in Supplementary Table S5, with additional centrality metrics.

We estimated the predicted number of taxonomic lineages for each level of sequencing power with rarefaction curves (Supplementary Fig. S2). MetaB18SV9 was the only sequencing approach that clearly reached the plateau of the rarefaction curve, but just for the 3–20 µm size fraction samples and because of a higher number of reads (Supplementary Fig. S2). While the MetaB16SV4V5 did not reach a clear plateau of the rarefaction curve, it was fairly close (Supplementary Fig. S2). Metagenomes were closer to the plateau of the rarefaction curve for protists than for prokaryotes (Supplementary Fig. S2).

### The effect of seawater filtered volume in marine microbial diversity, accounting for filter pore size

A considerable range of seawater volumes, from as low as 1 L up to 1000 L, was filtered using several pore sizes (whole water with 0.22 µm, or size fractions with 0.22–3 µm, 3–20 µm and >20 µm). Additionally, whole water cartridge membrane volumes from 1 L to 10 L were also compared for the metagenomes. Below, we consider the prokaryotes and protists results independently.

#### Prokaryotes

**Fig. 2:**
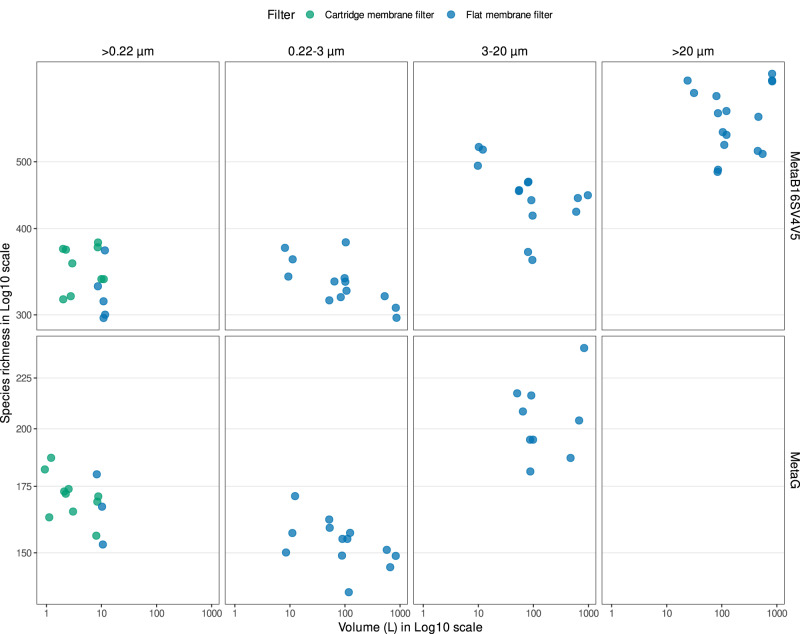
Overview of the prokaryotic species richness obtained. The grid divides the possible sequencing strategies in rows (MetaB16SV4V5 or metagenome) and the utilization of whole water (>0.22 µm) or size fractions (0.22–3 µm, 3–20 µm and >20 µm) in columns. Color distinguishes between flat and cartridge membrane filters. Within each grid unit, the prokaryotic species richness is plotted against volume, which ranges from 2.5 L to 1000 L.

In Fig. 2, we illustrate the number of prokaryotic taxonomic lineages obtained filtering the different seawater volumes, separated by filter type (cartridge membrane and flat membrane), pore sizes (whole water and size fractions) and sequencing strategy (MetaB16SV4V5 and metagenomes). The broad view highlights the absence of any clear effect of the filtered volume (1 L to 1000 L) on the number of prokaryotic taxonomic lineages obtained, independently of the filter type and sequencing approach (MetaB16SV4V5 and metagenome) for whole water and the fraction 0.22–3 µm. For flat membrane filters the number of prokaryotic taxonomic lineages increased with increasing pore size, but not with increasing volume (Fig. 2). Furthermore, the rarefaction curves of MetaB16SV4V5 revealed that the number of prokaryotic taxonomic lineages were consistently divided by size fractions (Supplementary Fig. S2), but not by volume (Supplementary Fig. S3). The same differences were observed for metagenomes, but only at a lower number of reads (Supplementary Fig. S3). The number of prokaryotic taxonomic lineages obtained after each sample are available in Supplementary Table S6.

**Fig. 3:**
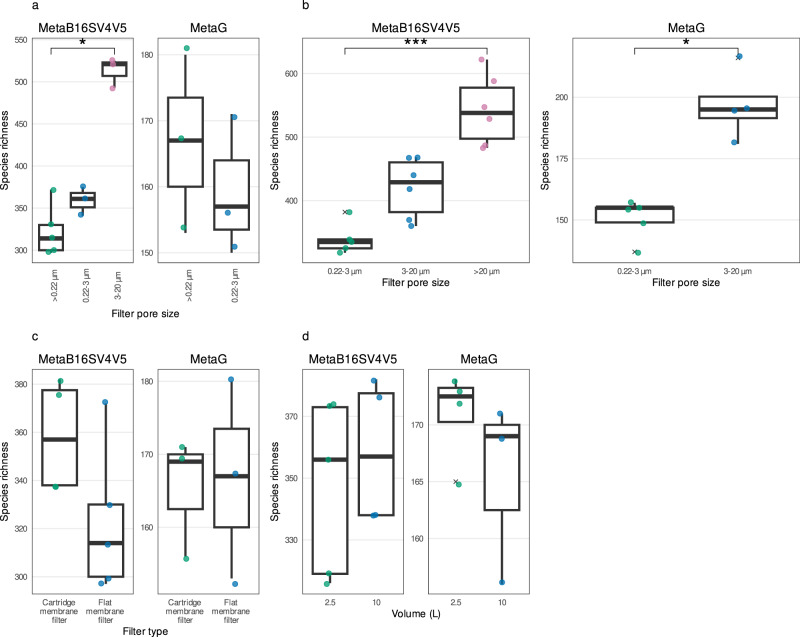
Detailed comparison of the prokaryotic species richness across methodological variables. **a** Comparison for whole water (>0.22 µm), 0.22–3 µm and 3–20 µm size fractions for the same volume (10 L) and filter (flat membrane), for MetaB16SV4V5 (left) and metagenomes (right). Note that metagenomes didn’t include samples in 3–20 µm size fraction in (**a**). **b** Comparison for size fractions (0.22–3 µm, 3–20 µm and > 20 µm size fractions) for the same volume (100 L) and filter (flat membrane), for MetaB16SV4V5 (left) and metagenomes (right). Note that metagenomes didn’t include samples in >20 µm size fraction in (**b**). **c** Comparison for flat membrane vs cartridge membrane, for the same volume (10 L) and whole water (>0.22 µm), for MetaB16SV4V5 (left) and metagenomes (right). **d** Comparison between 2.5 L (single filter) and 10 L (four 2.5 L filters pooled together), using the same filter (cartridge membrane) and whole water (> 0.22 µm), for MetaB16SV4V5 (left) and metagenomes (right). All panels illustrate the species richness obtained for each sample (point). To help the reader compare the variables, we added boxplots on top of the points. Significance was determined using either Mann–Whitney test for two independent groups, or Kruskall–Wallis for more than two independent groups, followed by a post-hoc Dunn test, if needed. Significance was illustrated with the symbols: *p* > 0.05 (empty); *p* < 0.05 (*); *p* < 0.01 (**); and *p* < 0.001 (***).

To clarify the effect of using either a single filter (whole water) or several consecutive filters (size fractions) in the number of prokaryotic taxonomic lineages obtained, we directly compared samples of the same filter (membrane) and volume (10 L). For MetaB16SV4V5, whole water (>0.22 µm) and 0.22–3 µm size fraction samples presented a similar number of prokaryotic taxonomic lineages (Fig. 3a) and both presented fewer prokaryotic taxonomic lineages than the 3–20 µm size fraction (Fig. 3a). Accordingly, the statistical test indicated significant differences in the species richness obtained after > 0.22 µm, 0.22–3 µm and 3–20 µm (*p* < 0.05, Kruskal–Wallis), more specifically, between >0.22 µm and 3–20 µm size fractions (*p* < 0.05, *post-hoc* Dunn test). On the metagenomes side, for the same comparison, there were no appreciable differences in the number of prokaryotic taxonomic lineages (Fig. 3a) and they were not significant (*p* > 0.05, Kruskal–Wallis). Details on the above-mentioned statistical tests are available in Supplementary Table S7.

Following the same reasoning, we compared the size fractions of 0.22–3 µm, 3–20 µm and >20 µm, using the flat membrane filter, which revealed an increase in the prokaryotic species richness with increasing pore size, for both MetaB16SV4V5 and metagenomes (Fig. 3b). In fact, the median number of prokaryotic taxonomic lineages obtained by MetaB16SV4V5 increased significantly from 335 (0.22–3 µm) to 429 (3–20 µm) and 538 (>20 µm) (*p* < 0.05, Kruskal–Wallis, Fig. 3b), more specifically between 0.22–3 µm and > 20 µm size fractions (*p* < 0.05, *post-hoc* Dunn test). Similarly, metagenomes increased the median number of prokaryotic taxonomic lineages from 155 (0.22–3 µm) to 195 (3–20 µm) (Fig. 3b), which was also significant (*p* < 0.05, Mann–Whitney). Details on the above mentioned statistical tests are available at Supplementary Table S7. Please note that for metagenomes in Fig. 3b there are no samples for the >20 µm size fraction because some samples were lost due to insufficient DNA for sequencing, while some samples that were successfully sequenced were later discarded due to low number of reads (below 10 000 reads, for a list of discarded samples in the rarefaction step see Supplementary Table S4). The overview of prokaryotic species richness was overall consistent and supported by the rarefaction curves because the different size fractions had similar levels of alpha diversity, while the same did not apply for volume (Supplementary Figs. S2 and S3).

To verify the specific effect of the filter (cartridge membrane or flat membrane), filters were compared for the same volume (10 L) and pore size (whole water, 0.22 µm) (Fig. 3c). The number of prokaryotic taxonomic lineages identified by MetaB16SV4V5 was higher for the cartridge membrane filter (Fig. 3c), but this difference was not very appreciable, because the range of values for the flat membrane filter included almost the entire range of values from the cartridge membrane filter. More specifically, the number of prokaryotic taxonomic lineages obtained with the flat membrane filter ranged from 297 to 372 (median = 314, IQR = 30, *n* = 5), while for the cartridge membrane filter, this number ranged from 332 to 382 (median = 357, IQR = 40, *n* = 4). Accordingly, the difference between the number of prokaryotic taxonomic lineages between cartridge and flat membrane filters was not significant (*p* > 0.05, Mann–Whitney). Metagenomes provided an equivalent number of prokaryotic taxonomic lineages between either filter (Fig. 3c) and the differences were not significant (*p* > 0.05, Mann–Whitney). Although we compared cartridge and flat membrane filters under the same volume (10 L), the cartridge membrane filters reached 10 L by pooling together four cartridge membrane filters of 2.5 L. However, the single 2.5 L cartridge membrane filter and 10 L pooled from four cartridge membrane filters of 2.5 L obtained an equivalent number of prokaryotic taxonomic lineages, without significant differences (*p* > 0.05, Mann–Whitney) for either sequencing approach (Fig. 3d). Details on the above mentioned statistical tests are available at Supplementary Table S7.

**Fig. 4:**
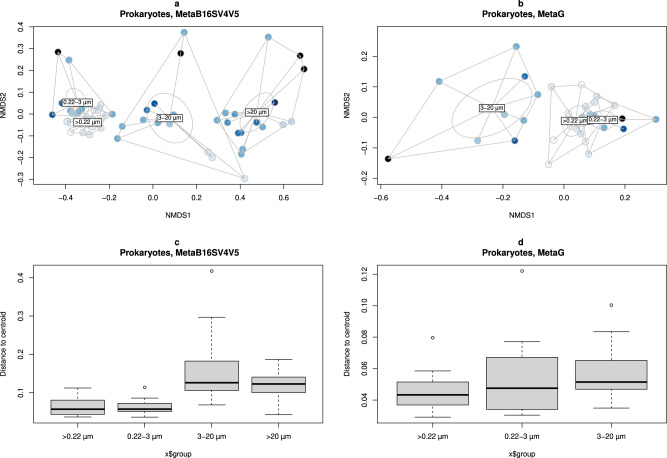
Prokaryotic community composition overview. MDS ordination of dissimilarity values (Bray–Curtis) for the prokaryotic community obtained in each sample. Samples were colored by volume and grouped by whole water (>0.22 µm), 0.22–3 µm, 3–20 µm and >20 µm size fractions. Division by (**a**) MetaB16SV4V5 and (**b**) metagenomes. Additionally, boxplots represent the distance to centroids of samples within each size fraction, divided by (**c**) MetaB16SV4V5 and (**d**) metagenomes. Note that metagenomes didn’t include the >20 µm size fraction. For details on missing replicates, we refer the reader to Supplementary Table S1.

The alpha diversity results for the full range of volumes and size fractions were consistent with beta diversity. For either MetaB16SV4V5 and metagenomes, whole water and 0.22–3 µm size fraction samples were clustered together in the nMDS analysis (Fig. 4a, b), followed by two other distinct clusters of the samples from the 3–20 µm and >20 µm size fractions. Additionally, the volume did not follow any clear direction in the ordination figures (Fig. 4a, b). PERMANOVA tests were made to support the ordination figures, with similar results for MetaB16SV4V5 and metagenomes. Specifically, both volume and size fractions significantly changed the community composition (*p* < 0.05, PERMANOVA), but this result should be interpreted with caution, because if the same test considers the division of samples by size fraction, then community composition did not change significantly across volume (*p* > 0.05, PERMANOVA). Details on the PERMANOVA statistical tests for prokaryotes are available at Supplementary Table S8. The variation within size fractions, measured by distance to centroid, further supported the clustering of prokaryotic community composition by size fractions (Fig. 4c,d, Supplementary Table S9).

**Fig. 5:**
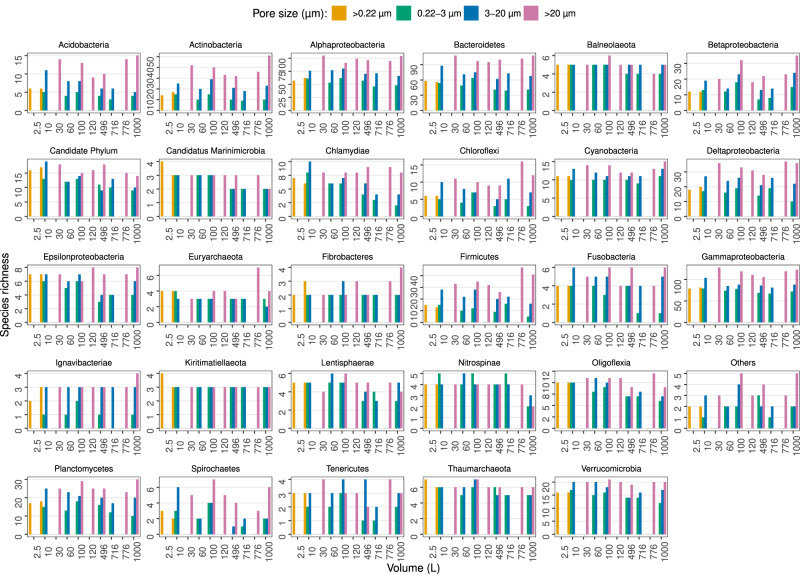
Prokaryotic species richness per taxonomic group, from MetaB16SV4V5. Each panel represents the species richness of a specific prokaryotic phyla or class for each volume (1–1000 L). Bar plots indicate species richness and are colored by pore size. The taxonomic group called “Others” includes all phyla that didn’t reach, at least, 100 taxonomic lineages across all samples, to avoid an excessive amount of uninformative, redundant panels. The taxonomic group called “Candidate Phylum” includes all phyla with candidate designation, except for candidate phyla Marinimicrobia.

**Fig. 6:**
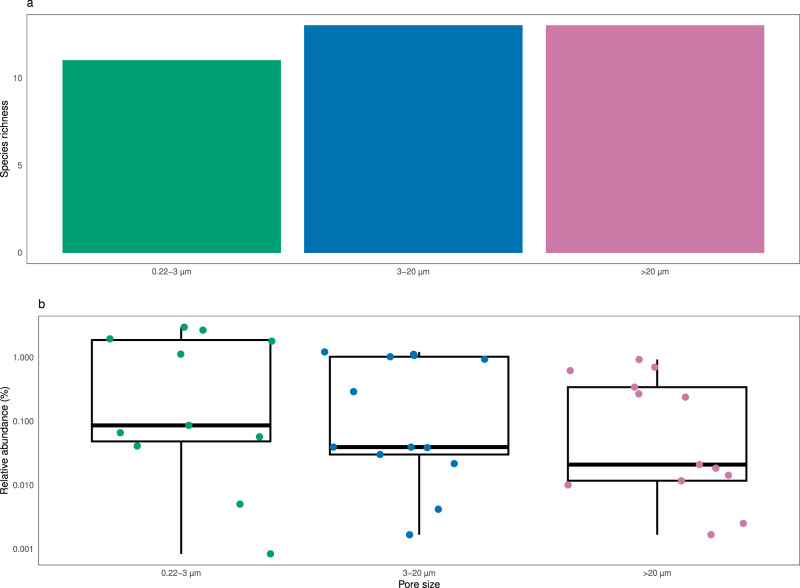
Detailed analysis of candidate phyla Marinimicrobia, from MetaB16SV4V5. **a** Number of candidate phyla Marinimicrobia taxonomic lineages and (**b**) relative abundance of the taxonomic lineages from (**a**). The values from (**a**) and (**b**) were compared for each size fraction (0.22–3 µm, 3–20 µm and > 20 µm) using the same volume (100 L) and filter (flat membrane).

The former alpha and beta diversity patterns could be the reflection of a restricted group of dominant taxa, instead of the entire microbial community. To verify possible differences due to taxonomy, the number of taxonomic lineages for each prokaryotic taxonomic group (see Materials and Methods) was compared against the volume and size fractions (Fig. 5). This comparison revealed that size fractions, and not volume, affected the species richness within high level taxonomic groups, for either MetaB16SV4V5 (Fig. 5), or metagenomes (Supplementary Fig. S4). A more detailed analysis revealed that prokaryotic species richness across volume changed differently depending on size fraction, as was the case for some major groups (Gammaproteobacteria, Alphaproteobacteria, Bacteroidetes, Deltaproteobacteria, Acidobacteria and Plantcomycetes) (Fig. 5). Although the metagenomes presented less taxonomic groups at phylum and class level, most were consistently separated by size fraction across volumes, like Gammaproteobacteria and Alphaproteobacteria, but some were not, like Betaproteobacteria and Thaumarchaeota (Supplementary Fig. S4). Note that we analyzed species richness within the selected taxonomic groups and not their relative abundance. To illustrate the difference, we plotted the number of taxonomic lineages attributed to Candidatus Marinimicrobia and their relative abundance for each size fraction at 100 L of volume (Fig. 6). The example from candidate phylum Marinimicrobia shows that even though it did not change the number of taxonomic lineages (Fig. 6a), their relative abundance decreased with increasing pore size of the size fractions (Fig. 6b). It is possible that finer differences exist at lower taxonomic levels for other phyla, but the full analysis of such possibilities goes beyond the scope of this study.

#### Protists

**Fig. 7:**
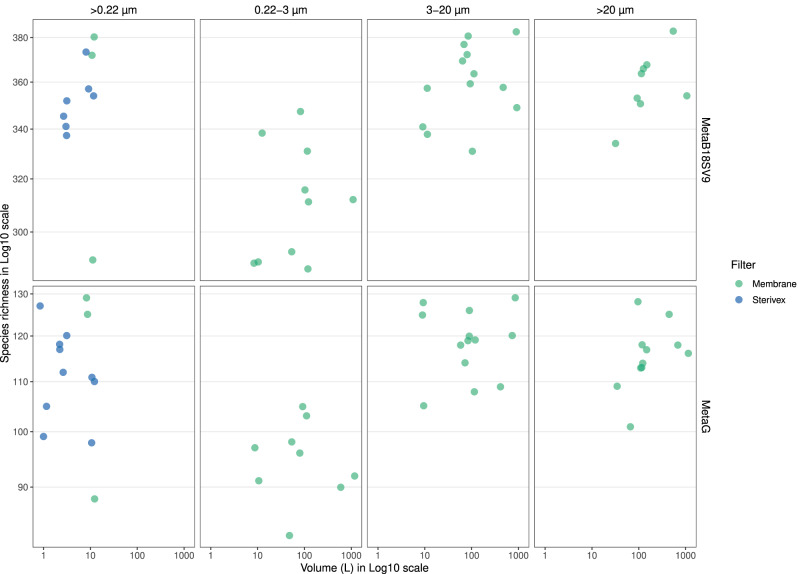
Overview of protist species richness obtained. The grid divides the possible sequencing strategies in rows (MetaB18SV9 or metagenome) and the utilization of whole water (>0.22 µm) or size fractions (0.22–3 µm, 3–20 µm and >20 µm) in columns. Color distinguishes between flat membrane and cartridge membrane filters. Within each grid unit, the protist species richness is plotted against volume. For details on missing replicates, we refer the reader to Supplementary Table S1.

Generally, the protist species richness was more affected by the pore size and by the utilization of consecutive filters, than by the volume (Fig. 7). More specifically, only whole water filtration (>0.22 µm) for MetaB18SV9 showed any appreciable change in the protist species richness from 2.5 L (median = 343, IQR = 6.75, *n* = 4) to 10 L (median = 348, IQR = 34.8, *n* = 12) (Fig. 7). However, for either MetaB18SV9 and metagenomes, there was no appreciable difference in the protist species richness from 10 L to 1000 L, within any of the size fractions (Fig. 7). Comparing pore sizes, whole water (>0.22 µm), 3–20 µm and >20 µm size fractions identified more protist taxonomic lineages than 0.22–3 µm size fraction samples (Fig. 7). The number of protist taxonomic lineages obtained after each sample are available at Supplementary Table S10. The higher impact of size fraction, rather than volume, on protist species richness was further supported by rarefaction curves (Supplementary Figs. S2 and S3), even though the size fractions were not as distinct from one another as they were with the prokaryotic data.

**Fig. 8:**
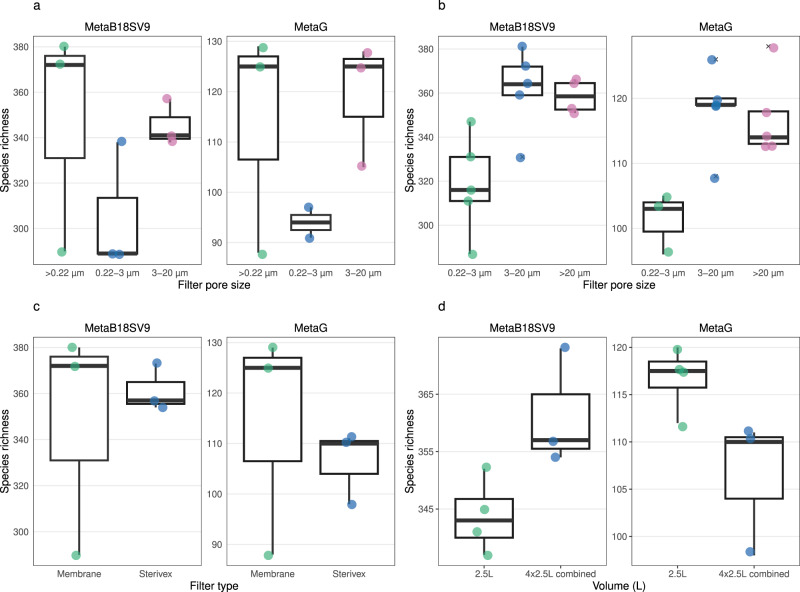
Detailed comparison of the protist species richness across methodological variables. **a** Comparison for whole water (>0.22 µm), 0.22–3 µm and 3–20 µm size fractions for the same volume (10 L) and filter (membrane), for MetaB18SV9 (left) and metagenomes (right). **b** Comparison for size fractions (0.22–3 µm, 3–20 µm and > 20 µm size fractions) for the same volume (100 L) and filter (membrane), for MetaB18SV9 (left) and metagenomes (right). **c** Comparison for flat membrane vs cartridge membrane, for the same volume (10 L) and whole water (>0.22 µm), for MetaB18SV9 (left) and metagenomes (right). **d** Comparison between 2.5 L (single filter) and 10 L (four 2.5 L filters pooled together), using the same filter (cartridge membrane) and whole water (> 0.22 µm), for MetaB18SV9 (left) and metagenomes (right). All panels illustrate the species richness obtained for each sample (point). To help the reader compare the variables, we added boxplots on top of the points. Significance was determined using either Mann–Whitney test for two independent groups, or Kruskall–Wallis for more than two independent groups, followed by a *post-hoc* Dunn test, if needed. Significance was illustrated with the symbols: *p* > 0.05 (empty); *p* < 0.05 (*); *p* < 0.01 (**); and *p* < 0.001 (***).

To verify the impact of using either whole water or size fractions, we compared these samples for the same volume (10 L) and filter (flat membrane). Both MetaB18SV9 and metagenomes had fewer protist taxonomic lineages in the 0.22–3 µm size fraction than in the whole water (>0.22 µm) or 3–20 µm size fraction (Fig. 8a). However, the range of the number of protist taxonomic lineages obtained for whole water included the range of values for both the 0.22–3 µm and 3–20 µm size fractions (Fig. 8a). More specifically, the number of protist taxonomic lineages obtained by MetaB18SV9 varied between 290 and 380 for the whole water, 289 and 338 for 0.22–3 µm size fraction, and 338 to 357 in 3–20 µm size fractions (Fig. 8a), which were not significantly different (*p* > 0.05, Kruskal–Wallis). The number of protist taxonomic lineages obtained by metagenomes varied between 88 and 129 for whole water, 91 and 97 for 0.22–3 µm, and 105 and 128 for 3–20 µm size fractions (Fig. 8a); these differences were also statistically non-significant (*p* > 0.05, Kruskal–Wallis). We note, however, the number of samples for the metagenome provide little support for the described differences in this specific comparison. Details on the above mentioned statistical tests are available in Supplementary Table S11.

Directly comparing the size fractions of 0.22–3 µm, 3–20 µm and >20 µm size fractions for the same filter (membrane) and volume (100 L), the 0.22–3 µm size fraction had fewer protist taxonomic lineages than the 3–20 µm and >20 µm size fractions (Fig. 8b), for either MetaB18SV9 and metagenomes. These differences were significant for the MetaB18SV9 (*p* < 0.05, Kruskal–Wallis), but not for the metagenomes (*p* > 0.05, Kruskal–Wallis). However, the significance of the test was not very strong and the *post-hoc* test for MetaB18SV9 was not significant for any combination of size fractions, after adjustment (*p* > 0.05, *post-hoc* Dunn test). Details on the above mentioned statistical tests are available in Supplementary Table S11.

To verify the specific effect of the filter type, cartridge and flat membrane filters were compared for the same volume (10 L) and pore size (whole water, >0.22 µm). The differences in the number of protist taxonomic lineages between cartridge and flat membrane filters were small (Fig. 8c) and not significant (*p* > 0.05, Mann–Whitney). However, the range of values was wider for the flat membrane filter than the cartridge membrane filter with the MetaB18SV9 approach (Fig. 8c). The number of protist taxonomic lineages within the replicates of flat membrane filters varied between 290 and 380 (difference of 90 taxonomic lineages), while in the cartridge membrane filters varied between 354 and 373 (difference of 19 taxonomic lineages) (Fig. 8c). For metagenomes, the values were equivalent between both types of filters (Fig. 8c). Please note that the cartridge membrane and flat membrane filters were compared at 10 L volume, but the cartridge membrane samples obtained 10 L by pooling together four cartridge membrane filters of 2.5 L together. For MetaB18SV9, the number of protist taxonomic lineages obtained after pooling four 2.5 L cartridge membrane filters was higher than using a single filter of 2.5 L (Fig. 8d), but not significant (*p* > 0.05, Mann–Whitney). However, this was not the same for the metagenomes, where the number of protist taxonomic lineages was equivalent and slightly higher for a single filter of 2.5 L (Fig. 8d), but also not significant (*p* > 0.05, Mann–Whitney). Details on the above mentioned statistical tests are available at Supplementary Table S11.

**Fig. 9:**
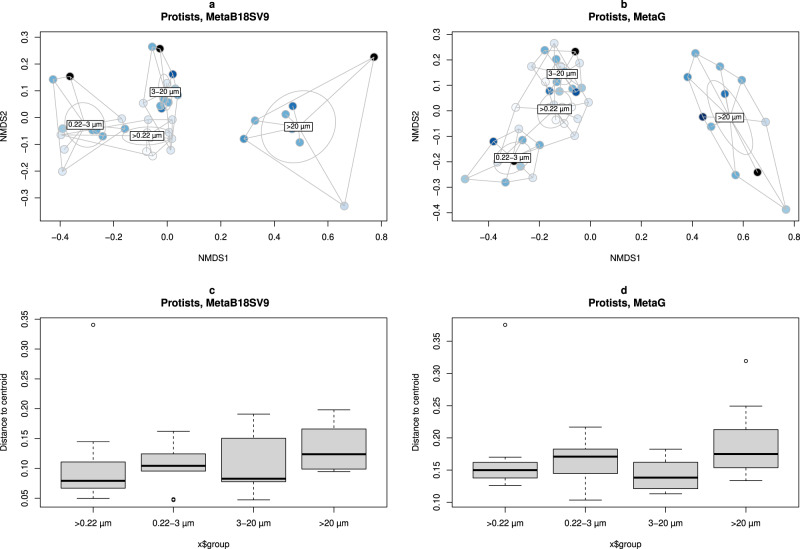
Protist community composition overview. MDS ordination of dissimilarity values (Bray–Curtis) for the protist community obtained in each sample. Samples were colored by volume and grouped by whole water (>0.22 µm), 0.22–3 µm, 3–20 µm and >20 µm size fractions divided by (**a**) MetaB18SV9 and (**b**) metagenomes. Additionally, boxplots represent the distance to centroids of samples within each size fraction, divided by (**c**) MetaB16SV4V5 and (**d**) metagenomes.

Beta diversity was consistent with species richness, because samples were grouped according to the pore size of the filter (Fig. 9a, b). Samples from smaller pore sizes (whole water, 0.22–3 µm and 3–20 µm) were near each other, while the samples for >20 µm size fractions were distant from the remaining, in either MetaB18SV9 and metagenomes (Fig. 9a, b). This was further supported by the significant results of PERMANOVA for the volume and size fractions independently (*p* < 0.05, PERMANOVA), but once they were considered together the effect on community composition was no longer significant (*p* > 0.05, PERMANOVA). Note that the variable for size fractions did not meet the homogeneity of variance pre-requisite of PERMANOVA (*p* > 0.05, betadisper). Details on the PERMANOVA statistical tests for protists are available in Supplementary Table S12. Additionally, a more detailed look into the betadisper results, i.e., a measure of distance to the centroid of samples within each size fraction, revealed that samples were very consistent within size fractions (Fig. 9c,d and Supplementary Table S13).

**Fig. 10:**
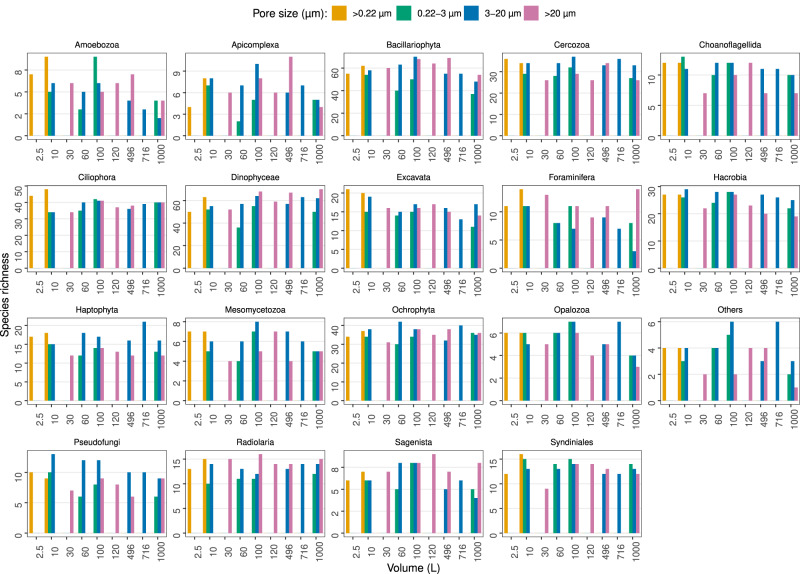
Protist species richness per taxonomic group, from MetaB18SV9. Each panel represents the species richness of a specific protist group for each volume (1–1000 L). Bar plots indicate species richness and are colored by pore size. Selected taxonomic groups follow a “fake rank” manually curated to highlight groups of interest, with the less representative groups merged into “Others” designation.

The taxonomic analysis of MetaB18SV9 didn’t reveal clear relations between the number of protist taxonomic lineages and volume, although some groups like Dinophyceae did show a small increase in their species richness with increasing volume (Fig. 10). For several protist taxonomic groups the >20 µm size fraction consistently identified more taxonomic lineages, independently of the volume, for example, Dinophyceae, Bacillariophyceae and Foraminifera (Fig. 10). In contrast, other groups were more prevalent in the 3–20 µm size fraction, like Cercozoa, Hacrobia and Haptophyta (Fig. 10). Several groups did not seem to favor any specific size fraction, like Excavata or Syndinales (Fig. 10). In the metagenomes, from 10 L to 1000 L, some groups had more protist taxonomic lineages in the >20 µm size fraction, like Bacillariophyceae, or fewer, like Hacrobia (Supplementary Fig. S5). Additionally, the metagenomes did not reveal any specific taxonomic group that increased the number of protist taxonomic lineages with increasing volume (Supplementary Fig. S5).

## Discussion

### The effect of methodological variation in common seawater sampling protocols

This study directly compared the seawater sampling methodologies used in major sampling campaigns of the global ocean for marine microorganisms—Tara Oceans [10], Malaspina [9], Ocean Sampling Day [13] and European Marine Omics Biodiversity Observation Network [16]. Our inter-comparison includes cartridge and flat membrane filters, by whole water filtration (single filter), or by size fractionation (serial water filtration of three filters of different pore sizes). The volumes filtered through cartridge membrane filters ranged from 1 L to 2.5 L, with an additional 10 L sample resulting from pooling together 4 samples of 2.5 L. This pooling step is common practice in several laboratories, since cartridge membrane filters are not practical for large volumes of seawater [62]. Such studies require usage of flat membrane filters instead, which in our case served for filtration from 10 L to 100 L, and for pooling 100 L samples into even larger volumes, up to 1000 L.

Our results clearly showed that pore size was the only methodological variable that significantly affected the description of microbial communities in terms of alpha and beta diversity. This was more evident for protists, as expected [39, 40], and underpins the reason why some expeditions, like Tara Oceans, used size fractionation [63]. Furthermore, studies have shown that pre-filtration steps and size fractions do, in fact, change the perception of microbial diversity [39, 64]. From a taxonomic point of view, most taxonomic groups could be identified in all size fractions. This was most surprising for the prokaryotes, where the >20 µm size fraction consistently had more taxonomic lineages, indicating that several taxonomic lineages were specifically found in that size fraction. One possible explanation for the identification of taxonomic lineages specific to the >20 µm size fraction is that those prokaryotes were attached to particles, or to the filter material itself. Considering that the turbidity of the water was very low, the only particles plausible for the prokaryotes to attach to would be the protists or other cell debris, including aggregates. Thus, we suggest that the prokaryotic taxonomic lineages specific to the large size fraction could be prokaryotes associated with microeukaryotes, colonial bacteria and/or specialized in colonizing larger particles. Given the presence of prokaryotes on > 20 µm size fractions and protists on 0.22–3 µm size fractions, we cannot rule out the possibility that extracellular DNA, besides actual cells, is retained in the filters, for example, by sorption [64]. However, the general picture is that free-living prokaryotes are identically identified in whole water (> 0.22 µm) and 0.22–3 µm size fraction, while particle-attached prokaryotes can be retained within larger pore size fractions (3–20 µm and >20 µm). This is consistent with previous studies that account for the effect of pre-filtration on prokaryotic diversity with 16 S rRNA gene sequencing [65]. Protists also follow the same general picture described in previous studies [40], with contamination between smaller size fractions, for example, because of cell fragments. In this study, either biological group was most unique in composition at >20 µm size fraction. Notwithstanding, we highlight that it was unexpected to find more prokaryotic and protist taxonomic lineages in the > 20 µm size fraction than in whole water, which cannot be fully explained by our experimental design and should be addressed in future work.

The patterns related to pore size were independent of the filtered volume. In fact, our work demonstrates that the volume of filtered water does not affect species richness and beta diversity of the analyzed sample. In other words, collecting more water, i.e., more cells, did not translate into more prokaryotic or protist taxonomic lineages, nor significant variations in community dissimilarity or different high level taxonomic composition. Additionally, it is noteworthy that we compared both 1 L to 10 L after whole filtration by cartridge membrane, and 10 L to 1000 L after size fractionation by three consecutive flat membrane filters, which exceeds by far the volumes filtered in most marine microbial ecological studies (usually up to 100 L, e.g., [7]), and is a higher range than previous studies on the effect of filtered volume and pre-filtration [65]. Regarding the utilization of cartridge membrane with 2.5 L or 10 L as a result of pooling together 4 samples of 2.5 L, the diversity metrics used were similar and the differences in community composition were close to zero. However, for specific taxonomic groups, the general rule does not necessarily apply. For example, the number of taxonomic lineages attributed to Dinophyceae increased with volume, even though it was a small increase.

Generally, we can argue that, except for pore size, there is little to no difference between protocols, and that the small differences can be due to stochastic events. Notwithstanding, we note that our analysis was mainly based on quantitative estimations of diversity and in specific situations, based on the study design and question, seemingly unimportant differences from a quantitative point of view might be relevant. For example, if the objective was focused on the candidate phylum Marinimicrobia, which is abundant in several marine environments and can play roles in marine biogeochemical cycles [66], it could be relevant to know that even though we can obtain a similar number of taxonomic lineages for each size fraction, their relative abundance decreases with size fraction. The mechanism to justify the higher relative abundance of candidate phylum Marinimicrobia on 0.22–3 µm size fraction is unknown to us. Despite that, we were expecting to find more abundant members of this candidate phylum in metagenomes, based on previous work comparing 16S rRNA gene amplicon sequencing and metagenomes from Arctic seawater samples [67]. Instead, candidate phylum Marinimicrobia was rare in the metagenome data (relative abundance below 0.1%). Besides this particular example, we did not explore the taxonomy further, leaving this challenge to future research.

### Consistency between sequencing strategies

In this study, we compared the above mentioned protocol variations in seawater sampling with distinct sequencing strategies. Specifically, amplicon sequencing of the V4-V5 hypervariable regions of the 16 S rRNA gene, and of the V9 hypervariable region of the 18 S rRNA gene, based on well-established primers [22, 42–45]. Total DNA shotgun sequencing was also included, which is a gene-untargeted approach lacking an amplification step and thus resulting in a lower number of single gene reads that can be used to determine microbial diversity, but is not affected by primer bias, e.g., Brown et al. [68]. The metagenome derived taxonomic lineages were further divided into prokaryotes and protists. Although the analyses were independently performed for each of the above mentioned groups, we were able to see that results were consistent between different sequencing strategies. The practical difference was on the number of taxonomic lineages, which was lower for prokaryotes and protists under metagenomes, while the relative difference between methodological variables tested were similar. Few exceptions include the comparison between using a single filter of 2.5 L or using 10 L (4 pooled filters of 2.5 L), where amplicon based approaches identified more taxonomic lineages in the pooled approach, while the metagenomes identified more taxonomic lineages in the single filter with 2.5 L. We note, however, that the differences were small in either situation. Collectively, it is clear that the methodological variables pose similar effects on observed species richness and beta diversity independently of sequencing strategy. Regarding taxonomy, metagenomes obtained lower resolution at high-level analysis than what was obtained for the amplicon based approaches, for either prokaryotes or protists. This lower resolution was a consequence of lower number of SSU gene reads overall, and not any of the sampling methodological variables tested. This was expected from previous studies where similar samples were compared for amplicon and metagenome based approaches, e.g., [67].

### Strengths and limitations of the EMOSE dataset

This dataset is publically available (see Data Availability section) and contains samples from more methodological variables than the ones presented in this work. For example, we did not use the samples from amplicon sequencing results of the 16 S rRNA gene divided by reads length at the library preparation stage. Another variable which we did not consider was the sequencing machine (HiSeq or MiSeq). A recent study compared replicates from HiSeq and MiSeq platforms, after 16S rRNA gene amplicon sequencing, and found differences in community composition [69]. We note, however, that the aforementioned study focused on the coral microbiome, while ours is focused on seawater microbial communities.

Due to the high number of different variables tested in this study, the EMOSE dataset has hundreds of samples, which is a significant advantage over up-to-date studies, but also has some limitations. Firstly, it might be complex to use the data available due to the high number of variables. Secondly, the high number of samples is a result of many variables tested, not of many replicates (*n* = 3, wherever possible). However, it should be noted that keeping the same number of replicates across all variables was not possible, due to on-site methodological and logistic impossibilities, including the effort and time needed to filter 100 L of seawater using three different filters. Some limitations are all the more understandable when we take into account the fact that, as far as we know, the presented dataset assesses the effect of filtration of several thousands of liters of seawater carried out for the first time on such a scale during a one-day/one-place campaign.

We decided to compromise with a traditional, easy to understand, rarefaction procedure, but we are aware of its limitations and possible implications. More specifically, rarefaction results in the loss of valid reads and of valid samples and does not account for the compositional nature of high-throughput sequencing data [70, 71]. Additionally, another study that directly compared seawater sampling strategies suggested not to standardize the sequencing results [34]. Some alternatives solve the normalization of compositional data without the need of removing valid reads, for example, centered-log ratio transformation [71]. However, alternative normalization procedures can make interpretation more difficult, for example, by giving negative values of diversity, and can be harder to understand than rarefaction, which is common practice between peers and relatively easy to interpret. We also note that the differences in sequencing depth between samples were considerably large in some cases and there is not a single ‘best’ normalization tool to solve that problem.

Regarding the statistical analysis of species richness, we used non-parametric tests that compare distributions and, as such, they should be accompanied by median and interquartile information, which we illustrated by means of boxplots. Although few replicates were used in some comparisons, which compromises confidence in data distributions [72], our analyses clearly demonstrate the impact of size fractionation on species richness for both metagenome and amplicon sequencing data. Furthermore, we acknowledge that using a single alpha diversity metric (species richness) might miss some tendencies of the data, we believe this would not be the case for the Shannon index, but it could be for the Simpson index based on correlation analysis (Supplementary Fig. S1).

In addition to the undeniable scientific importance, the presented research has also significant practical meaning. Considering our results, especially in the case of research which, due to limited resources and time, cannot afford to filter large volumes of seawater, we recommend partial filtration. As we demonstrated, dividing the efforts of filtering, inter alia, 1000 L into 100 samples of 10 L (which could be divided into dozens of replicates for each variable), still accurately represents the site in question and does not negatively affect the statistical power of the tests. Compared with, for example, two samples of 500 L each, there would be no improvement in the biodiversity recognized, and the statistical analysis would be compromised. Importantly, the applicative nature of this recommendation mainly concerns studies focusing on aspects of seawater microbial diversity. For studies with other objectives, the reasoning might not apply, or apply differently.

## Conclusion

Our findings highlight that different seawater sample volumes (from 1 L to 1000 L) and the filter types did not affect the identified prokaryotic and protists species richness and beta diversity. In contrast, through serial filtration with membranes of different pore sizes, the size fractionation was a crucial factor for the results obtained. Furthermore, the use of whole water filtration (>0.22 µm) was generally equivalent to the 0.22–3 µm size fraction. This metabarcoding and metagenomic comparison of sampling protocols can help researchers to design their own sampling campaigns and to compare studies using different protocols. Even though we did a tremendous effort to address many different variables in protocols used by different campaigns, there is more to be tested and compared for the purpose of standardization of protocols in the future, for example, DNA extraction protocols.

## Supplementary information

Supplementary Figure S1

Supplementary Figure S2

Supplementary Figure S3

Supplementary Figure S4

Supplementary Figure S5

Supplementary Table S1

Supplementary Table S2

Supplementary Table S3

Supplementary Table S4

Supplementary Table S5

Supplementary Table S6

Supplementary Table S7

Supplementary Table S8

Supplementary Table S9

Supplementary Table S10

Supplementary Table S11

Supplementary Table S12

Supplementary Table S13

## Data Availability

All raw sequences from the EMOSE dataset, which includes all data used in this article, are available in the European Nucleotide Archive under the accession number ERP090011. The abundance tables are available at MGnify platform under accession number MGYS00001935. Note that both Versions 5 and 4.1 include all sequencing strategies (MetaB16SV4V5, MetaB18SV9 and metagenomes). The metadata for each sample database was recorded in PANGEA [71], but a cleaner version is available at Supplementary Table S2. The R scripts for all data manipulation, statistical tests and figures is available in github (https://github.com/pascoalf/Inter-comparison-of-marine-microbiome-sampling-protocols), no manipulation of data was done outside the scripts provided, except for manual curation of taxonomy, because it needs human experts’ evaluation.
