# Supplementary material for: Inter-comparison of marine microbiome sampling protocols
Source: ISME Commun. 2023 Aug 19;3:84. doi: 10.1038/s43705-023-00278-w (PMC10439934; doi:10.1038/s43705-023-00278-w)
Supplement: Supplementary file 1 — Supplementary Figure S1 [file 43705_2023_278_MOESM1_ESM.pdf]

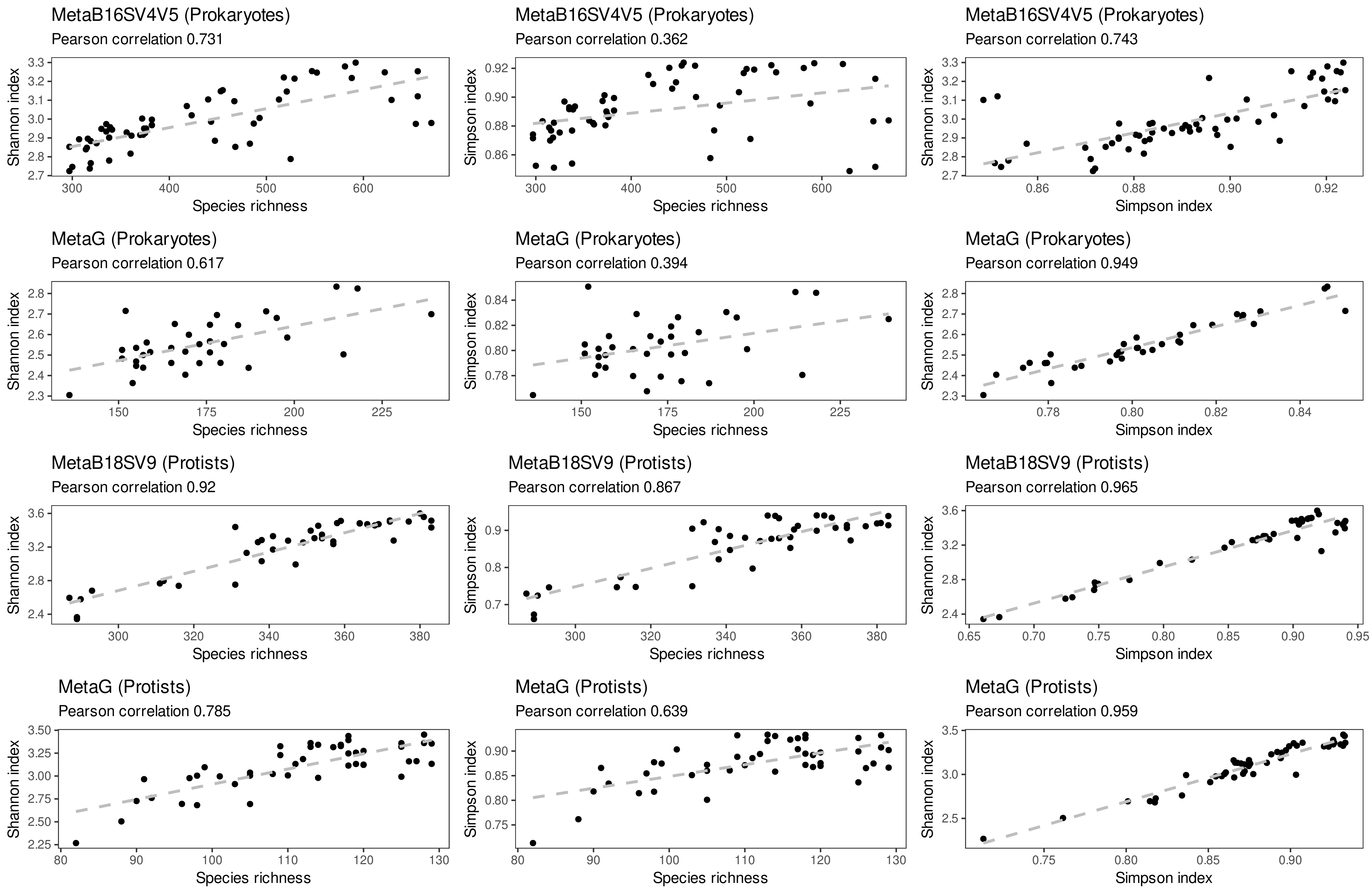
MetaB16SV4V5 (Prokaryotes)
Pearson correlation 0.731
Shannon index
Species richness
MetaB16SV4V5 (Prokaryotes)
Pearson correlation 0.362
Simpson index
Species richness
MetaB16SV4V5 (Prokaryotes)
Pearson correlation 0.743
Shannon index
Simpson index
MetaG (Prokaryotes)
Pearson correlation 0.617
Shannon index
Species richness
MetaG (Prokaryotes)
Pearson correlation 0.394
Simpson index
Species richness
MetaG (Prokaryotes)
Pearson correlation 0.949
Shannon index
Simpson index
MetaB18SV9 (Protists)
Pearson correlation 0.92
Shannon index
Species richness
MetaB18SV9 (Protists)
Pearson correlation 0.867
Simpson index
Species richness
MetaB18SV9 (Protists)
Pearson correlation 0.965
Shannon index
Simpson index
MetaG (Protists)
Pearson correlation 0.785
Shannon index
Species richness
MetaG (Protists)
Pearson correlation 0.639
Simpson index
Species richness
MetaG (Protists)
Pearson correlation 0.959
Shannon index
Simpson index
