# Supplementary figures and images for: Inter-comparison of marine microbiome sampling protocols
Source: ISME Commun. 2023 Aug 19;3:84. doi: 10.1038/s43705-023-00278-w (PMC10439934; doi:10.1038/s43705-023-00278-w)

**a**  
**MetaB16SV4V5**

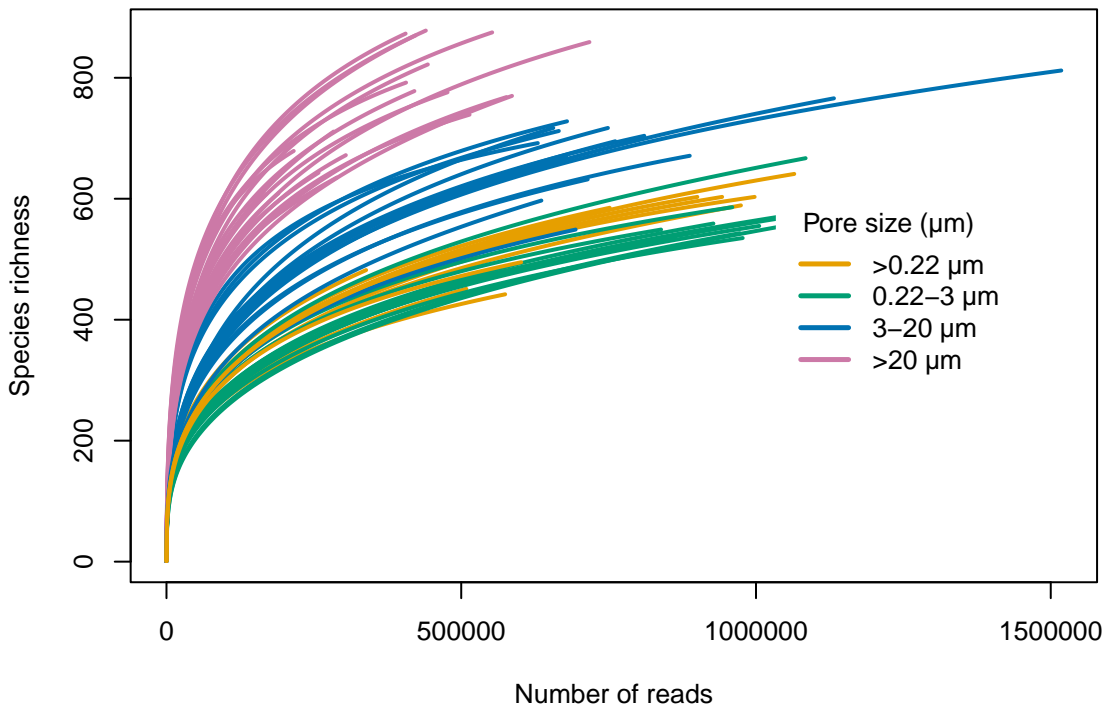

**b**  
**MetaB18SV9**

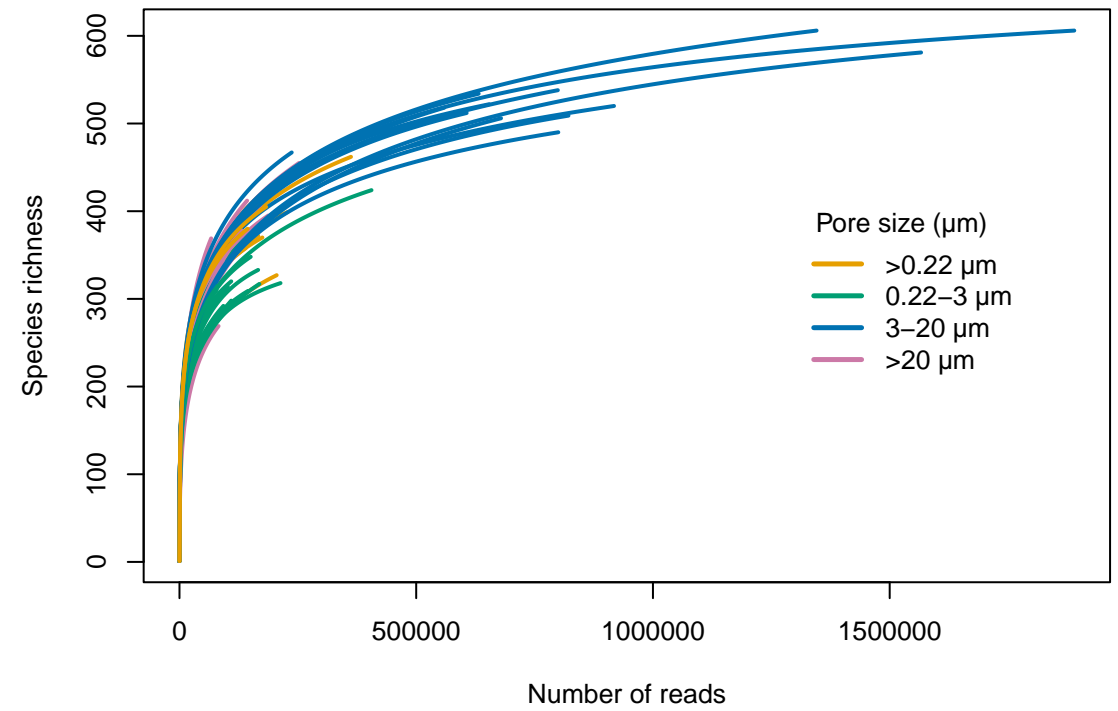

**c**  
**MetaG, Prokaryotes**

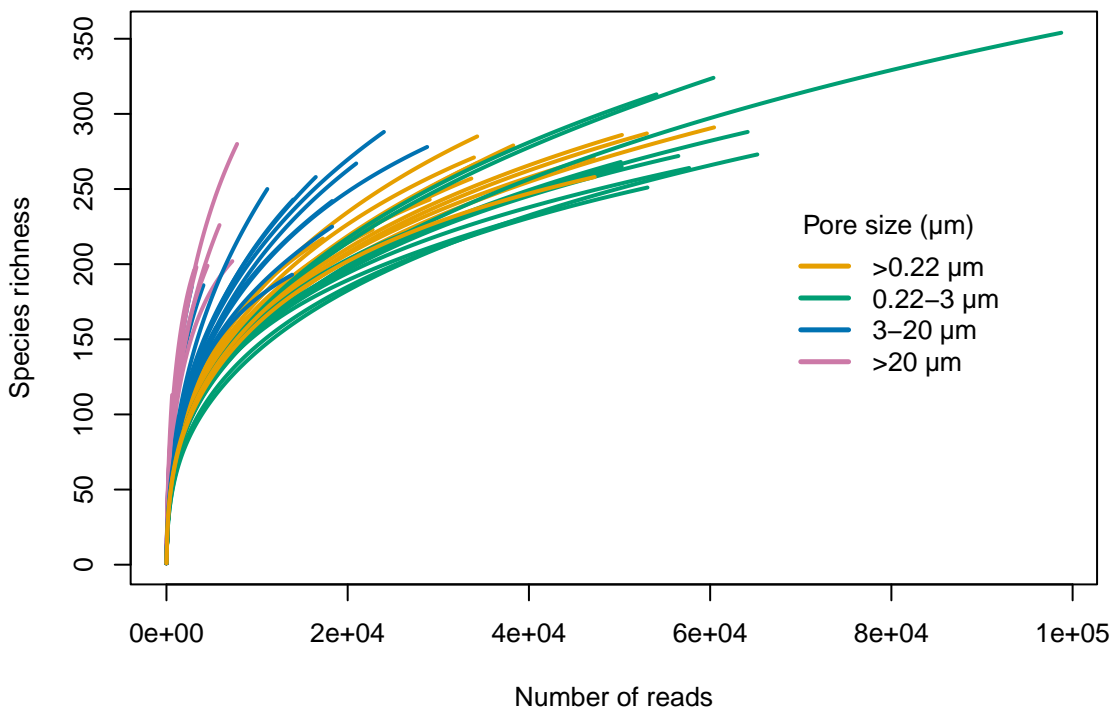

**d**  
**MetaG, Protists**

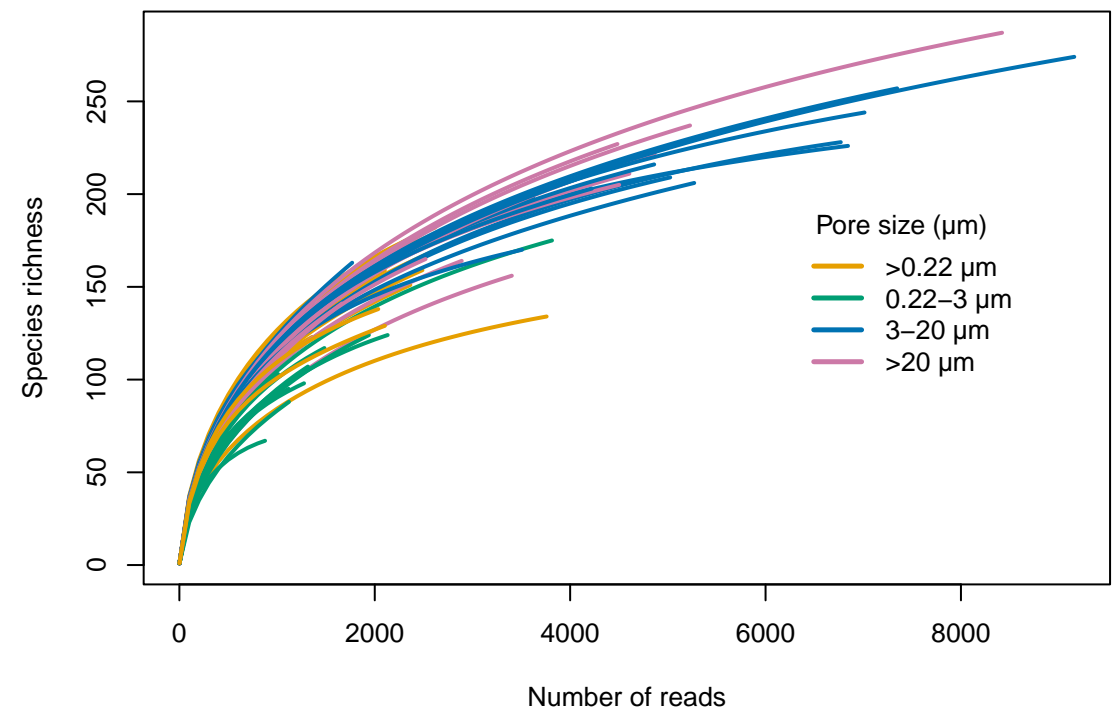

Supplement: Supplementary file 2 — Supplementary Figure S2 [file 43705_2023_278_MOESM2_ESM.pdf]

**a**  
**MetaB16SV4V5**

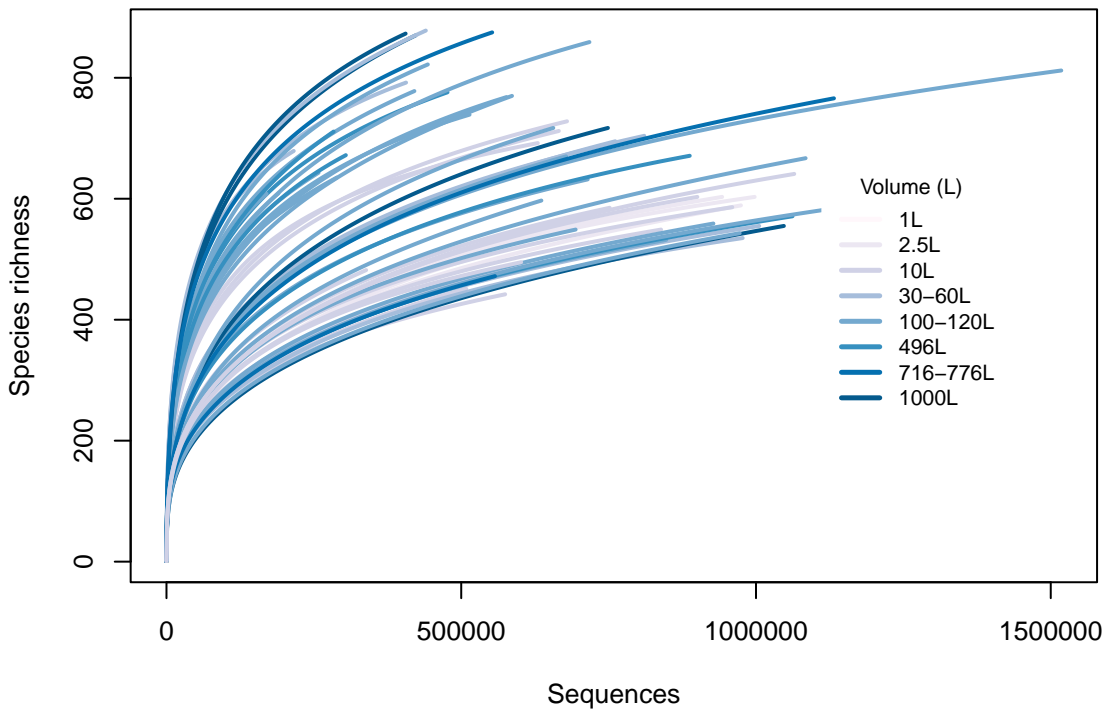

**b**  
**MetaB18SV9**

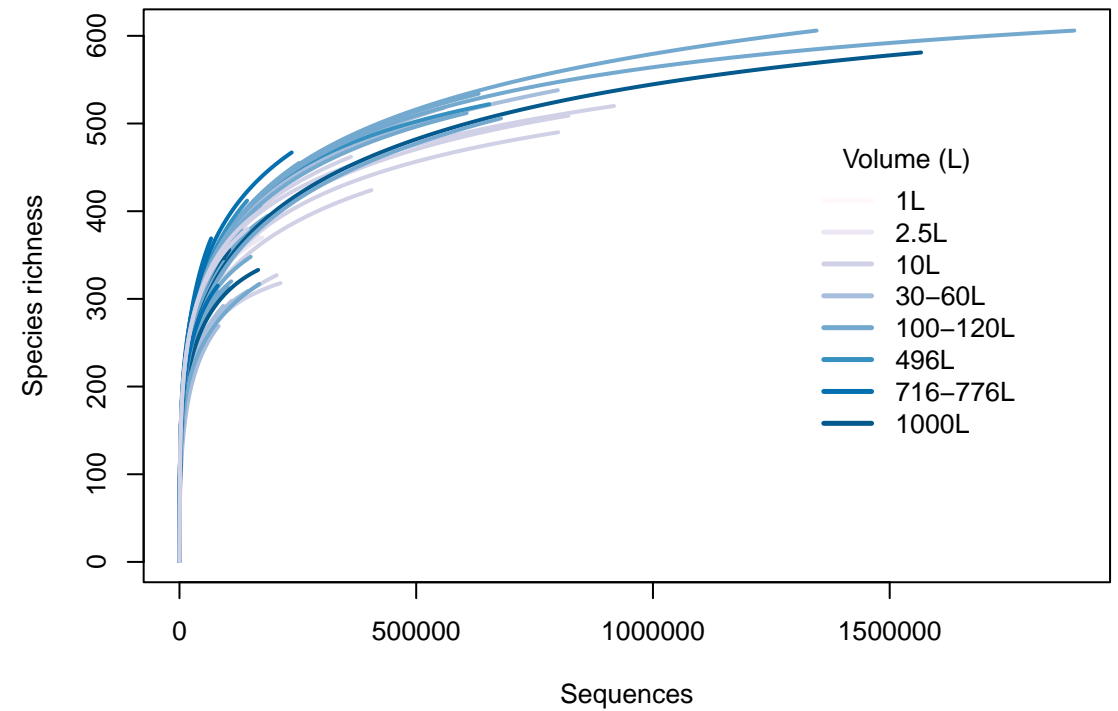

**c**  
**MetaG, Prokaryotes**

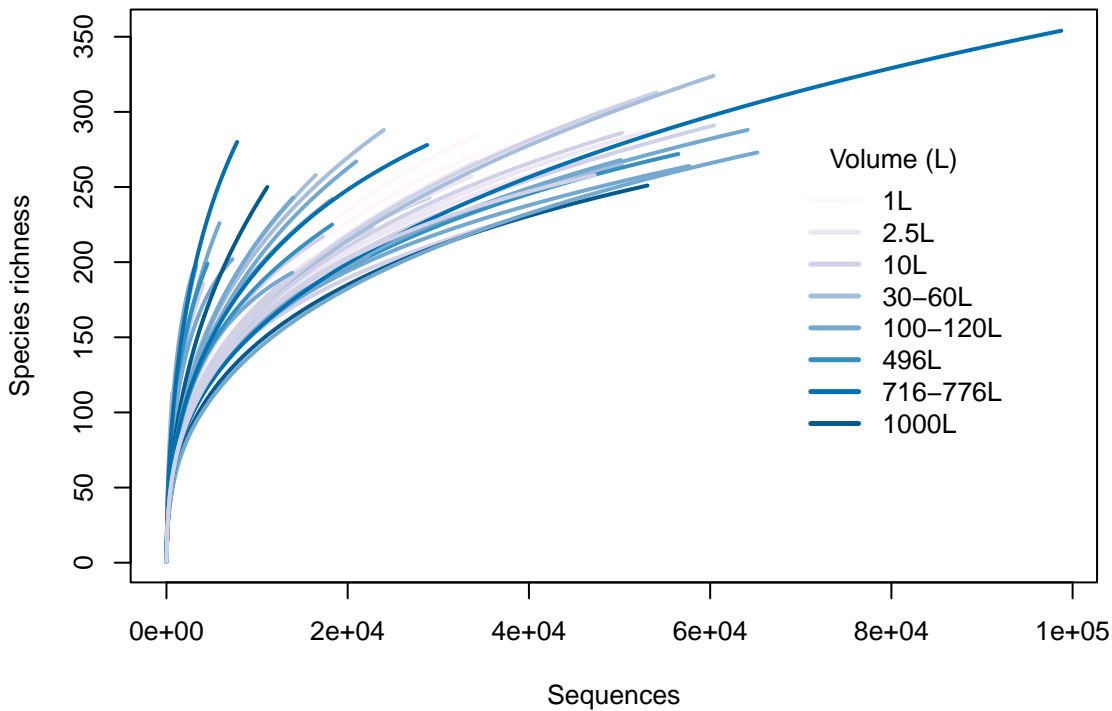

**d**  
**MetaG, Protists**

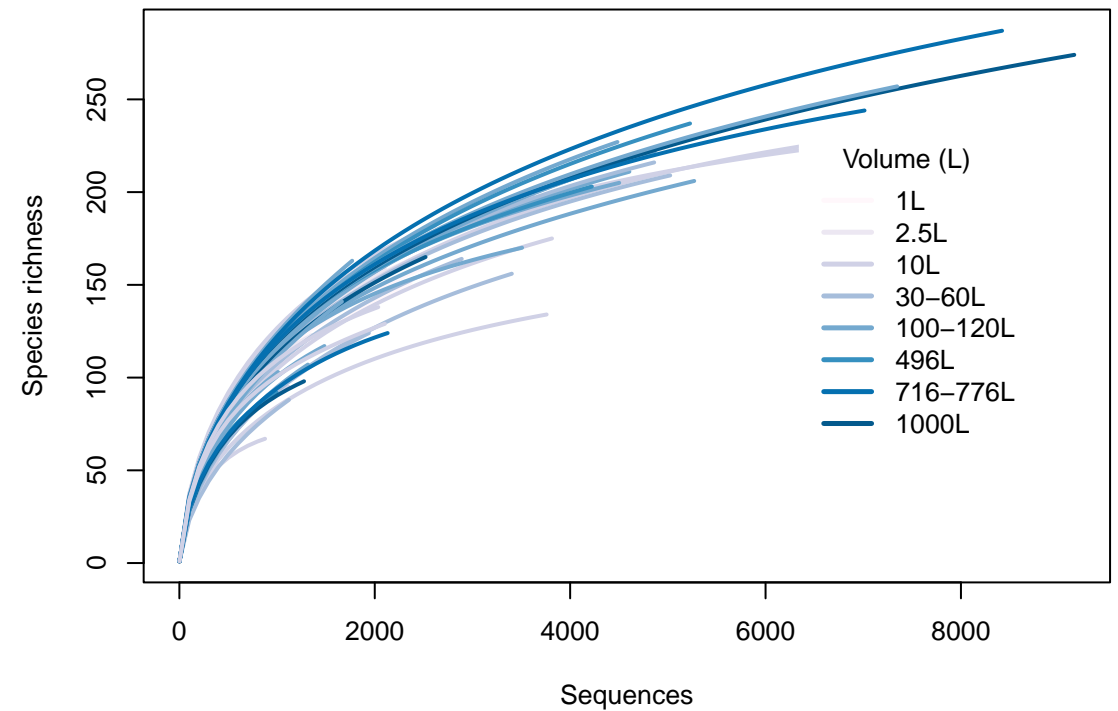

Supplement: Supplementary file 3 — Supplementary Figure S3 [file 43705_2023_278_MOESM3_ESM.pdf]

 >0.22 μm
  0.22–3 μm
  3–20 μm
  >20 μm

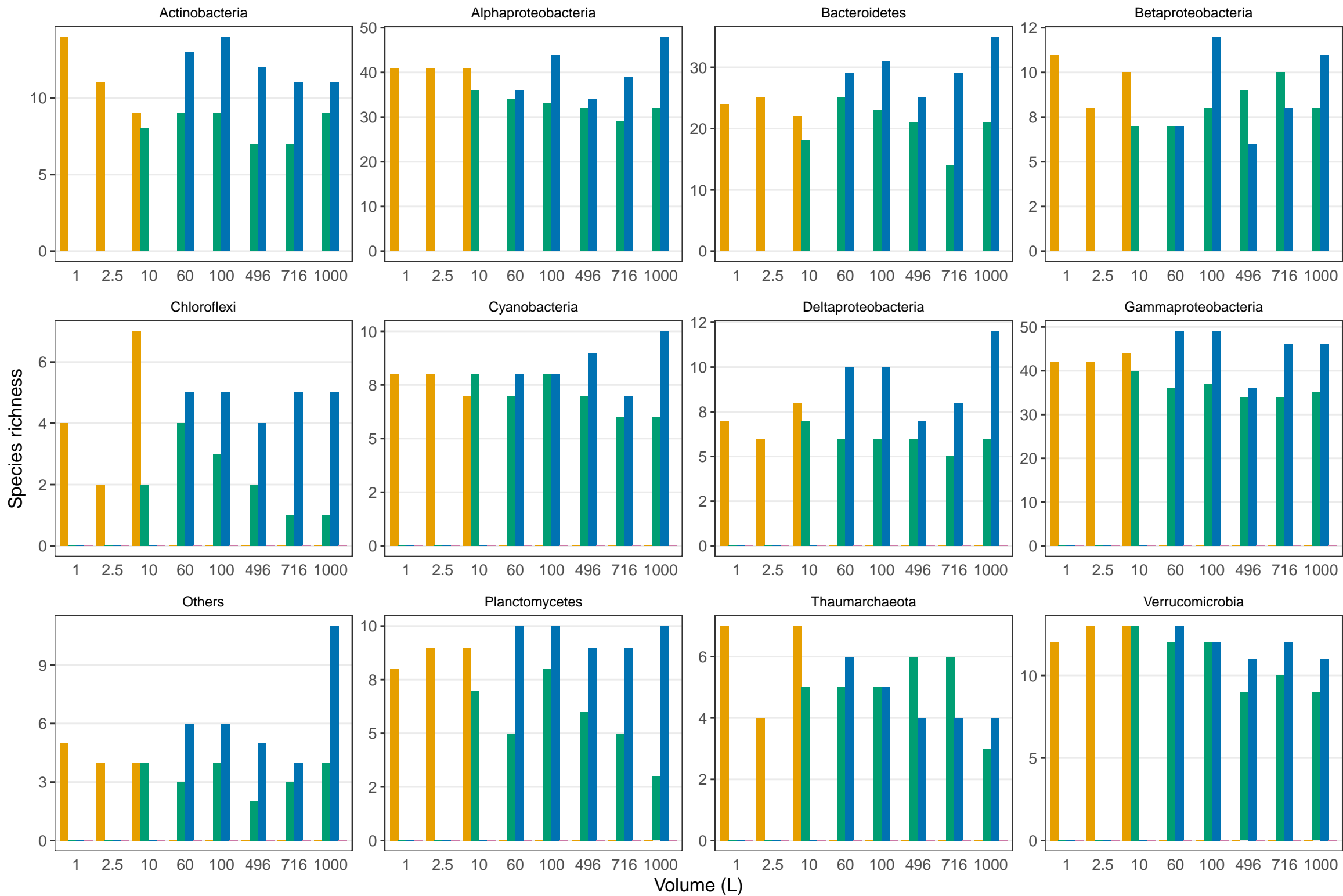

Supplement: Supplementary file 4 — Supplementary Figure S4 [file 43705_2023_278_MOESM4_ESM.pdf]
