# Supplementary material for: Inter-comparison of marine microbiome sampling protocols
Source: ISME Commun. 2023 Aug 19;3:84. doi: 10.1038/s43705-023-00278-w (PMC10439934; doi:10.1038/s43705-023-00278-w)
Supplement: Supplementary file 5 — Supplementary Figure S5 [file 43705_2023_278_MOESM5_ESM.pdf]

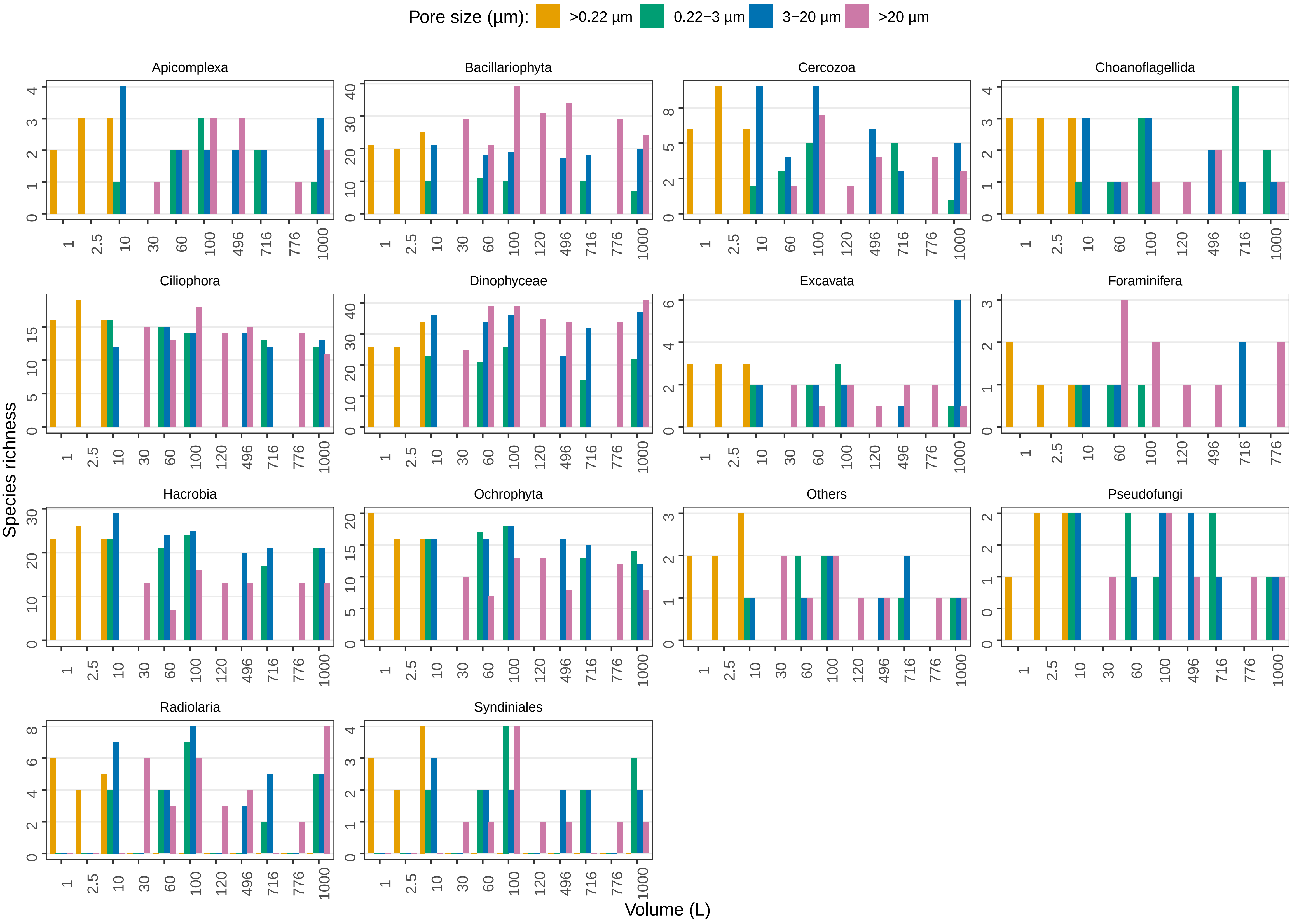
Pore size (µm):
>0.22 µm
0.22−3 µm
3−20 µm
>20 µm
Apicomplexa
Bacillariophyta
Cercozoa
Choanoflagellida
Ciliophora
Dinophyceae
Excavata
Foraminifera
Hacrobia
Ochrophyta
Others
Pseudofungi
Radiolaria
Syndiniales
Species richness
Volume (L)
